# Development of Polymer/Ionic Liquid/Polymer Trilayer Membranes for the Recovery of Blueberry Aroma and Fragrance Compounds from a Model Aqueous Solution by Pervaporation

**DOI:** 10.3390/membranes16080274

**Published:** 2026-08-17

**Authors:** Felipe Ramos, Daniela Guarda, Martina Gasset, David Aguilera, Thais González, Daniela Cerro, Andrea Plaza, Mickel Garrido, Fabian Cifuentes, Luis Pino-Soto, Julio Romero, R. Cabezas, Esteban Quijada-Maldonado, Gastón Merlet

**Affiliations:** 1 Laboratorio de Innovación en Soluciones Ambientales Integradas (LISAI), Departamento de Agroindustrias, Facultad de Ingeniería Agrícola, Universidad de Concepción, Vicente Méndez 595, Chillán 3812120, Chile; 2 Laboratory of Separation Processes Intensification (SPI), Department of Chemical Engineering, University of Santiago de Chile, Av. Libertador Bernardo O’Higgins 3363, Santiago 9170022, Chile; 3 Laboratorio de Innovación en Soluciones Ambientales Integradas (LISAI), Departamento de Recursos Hídricos, Facultad de Ingeniería Agrícola, Universidad de Concepción, Vicente Méndez 595, Chillán 3812120, Chile; 4 Laboratory of Membrane Separation Processes (LabProSeM), Department of Chemical Engineering, University of Santiago de Chile, Av. Libertador Bernardo O’Higgins 3363, Santiago 9170022, Chile; 5 Laboratorio de Ingeniería y Tecnología con Membranas (ITM), Departamento de Ingeniería Química, Facultad de Ingeniería, Universidad de Concepción, Edmundo Larenas 219, Concepción 4070386, Chile; 6 Departamento de Química Ambiental, Facultad de Ciencias, Universidad Católica de la Santísima Concepción, Alonso de Ribera 2850, Concepción 4090541, Chile

**Keywords:** pervaporation, ionic liquids, mass transfer, recovery aroma compounds, pervaporation membranes

## Abstract

This study investigates polymer/ionic liquid/polymer trilayer membranes for recovering aroma and fragrance compounds from a simplified synthetic model aqueous solution via pervaporation. The membranes featured organophilic polymer layers (PEBA and POMS) sandwiching an intermediate ionic liquid (IL) layer ([P_1444_][Tf_2_N] and [Bmim][Tf_2_N]), which were gelled with 12-hydroxystearic acid to ensure structural integrity. The trilayer architecture significantly enhanced separation performance compared with monophase membranes. The PEBA/[P_1444_][Tf_2_N]/PEBA configuration achieved the highest enrichment factors, reaching 1626 for hexanal and 963 for linalool. Meanwhile, the POMS/[P_1444_][Tf_2_N]/POMS system exhibited the highest selectivity relative to water. The introduction of the IL gel layer reduced overall mass transfer resistance while simultaneously suppressing water flux from 0.312 kg h^−1^ m^−2^ (in pure PEBA) to between 0.013 and 0.023 kg h^−1^ m^−2^. Overall, the results demonstrate that combining polymer matrices with gelled ILs in a trilayer design effectively optimizes mass transfer and selectivity, offering a promising strategy for the future valorization of aroma compounds from agro-industrial aqueous streams.

## 1. Introduction

Within the framework of a circular economy, the valorization of agro-industrial by-products has gained increasing relevance due to its potential to reduce environmental impacts while generating added economic value [1]. The blueberry industry, with global production of approximately 1.8 million tons in 2022, generates large amounts of liquid and solid waste during processing, particularly in exporting countries such as Chile [2]. These residues contain high-value aroma and fragrance (AF) compounds, including phenolics and anthocyanins, as well as volatile and semi-volatile aroma compounds such as alcohols, aldehydes, and terpenoids [3], which exhibit sensory, antioxidant, and antimicrobial properties [4]. Nevertheless, most of these compounds remain underutilized or are discarded during agro-industrial operations such as washing, juice production, and liquid effluent treatment, leading to both economic losses and environmental concerns associated with the discharge of organic-rich wastewaters [2].

The recovery of AF compounds from agro-industrial aqueous matrices is technically challenging due to their low concentrations and the complex composition of the mixtures. Conventional separation methods, including distillation, solvent extraction, and adsorption, present inherent drawbacks such as thermal degradation of thermosensitive compounds, solvent toxicity, and high operational and regeneration costs [5]. In this context, pervaporation has emerged as a promising and sustainable separation technology, enabling selective transport and evaporation of target compounds through dense membranes under a chemical potential gradient [6]. Pervaporation operates at moderate temperatures, avoids the use of organic solvents, and offers high selectivity, making it particularly suitable for the recovery of volatile and semi-volatile AF compounds from dilute aqueous solutions.

The performance of pervaporation processes is strongly governed by membrane material properties, including chemical affinity, free volume, and chain mobility. Polymeric membranes such as polyether block amide (PEBA) and polyoctylmethylsiloxane (POMS) have been extensively investigated for the separation of organic compounds due to their organophilic character, high permeability, and mechanical flexibility [7]. PEBA membranes consist of rigid polyamide hard segments and flexible polyether soft segments, which provide a compromise between mechanical stability, permeability, and selectivity toward moderately polar organic compounds. In contrast, POMS membranes, due to their siloxane backbone, show a strong affinity for nonpolar and weakly polar organic molecules and high permeability for hydrophobic aroma compounds.

In recent years, the incorporation of ionic liquids (ILs) into polymeric membranes has emerged as an effective strategy to tailor membrane affinity and enhance separation performance [8]. ILs such as [P_1444_][Tf_2_N] and [Bmim][Tf_2_N] are particularly attractive due to their negligible vapor pressure, high thermal and chemical stability, and strong solvating ability toward aromatic compounds [9,10]. Previous studies have demonstrated that these ILs can significantly improve the recovery of alcohols in vacuum-assisted perstraction systems. For instance, butanol fluxes up to 1.17 × 10^−3^ kg h^−1^ m^−2^ at 37 °C and a butanol/water selectivity of 6.73 have been reported. Moreover, resistance in series modeling revealed that the membrane accounted for approximately 98% of overall mass transfer resistance, emphasizing the critical role of membrane design in controlling separation efficiency [11].

Despite these advances, the direct incorporation of ILs into membranes often raises concerns regarding IL leakage and long-term stability under operating conditions. The immobilization of ionic liquids within a solid or gelled matrix to prevent leaching and to provide mechanical stability under pressure or vacuum gradients is a well-established strategy within the broader field of supported and gelled ionic liquid membranes [8,12,13,14,15,16,17]. Building on this established concept, and on the group’s own prior work on silicone-coated ionic liquid gel membranes [18], the present contribution consists of embedding the gelled IL as the intermediate layer of a polymer/IL-gel/polymer trilayer (sandwich-type) architecture, rather than as a single supported layer as in conventional supported ionic liquid membranes (SILMs). This configuration is intended to combine the mechanical integrity and leakage resistance already reported for gelled ionic liquid systems with the additional protection afforded by two outer dense polymeric layers, offering a more robust alternative to conventional single-support SILMs for pervaporation applications.

Accordingly, the main objective of this study is to develop and evaluate a pervaporation system based on selective three-layer membranes incorporating gelled ILs for the recovery of characteristic AF compounds from blueberry by-product model solutions. To achieve this objective, the following specific goals are pursued: (a) preparation of three- layer membranes comprising ILs stabilized by gelation and selective organophilic and hydrophobic membranes for AF compounds from blueberries; (b) evaluation of extraction capacities and transmembrane flux of AFs from model mixtures; (c) determination of the operating parameters affecting membrane separation performance; and (d) comparison of mass transfer resistance among the different membranes used in this study to identify the key factors responsible for performance differences.

## 2. Methodology

### 2.1. Materials and Reagents

Hexanal, benzyl alcohol, linalool, ethanol, hexanol, Z-3-hexen-1-ol, and E-2-hexen-1-ol were supplied by Merck^®^ (Santiago, Chile), with purities ranging from 96% to 99%. Model aqueous solutions were prepared by dissolving the target aroma and fragrance (AF) compounds in ultrapure water obtained from a PureLab^®^ Classic system (ELGA LabWater, High Wycombe, UK). The model solution was designed as a simplified synthetic aqueous matrix containing aroma and fragrance compounds representative of blueberry processing. Each compound was adjusted to an initial concentration of 1000 ppm, as reported in previous studies [19], to enable a controlled comparative evaluation of membrane transport. Therefore, the model solution was not intended to quantitatively reproduce the complete composition of a specific industrial blueberry-washing effluent. Table 1 summarizes the physicochemical properties of the compounds used in this study [20].

The present work establishes a baseline evaluation of membrane performance using pure standards of the target aroma and fragrance compounds, prior to the progressive incorporation of additional matrix constituents typically found in agricultural residues (e.g., sugars, organic acids, and suspended solids) and, ultimately, real residual streams. It is further anticipated that a pretreatment step, such as ultrafiltration, could be applied to the raw residue to reduce suspended-solid content and mitigate potential membrane fouling prior to the pervaporation stage.

The ILs 1-butyl-3-methylimidazolium bis(trifluoromethylsulfonyl)imide [Bmim][Tf_2_N], and tributylmethylphosphonium bis(trifluoromethylsulfonyl)imide [P_1444_][Tf_2_N] were supplied by Iolitec^®^ Heilbronn, Germany. These specific ILs were selected based on prior thermodynamic simulation work using COSMOthermX^®^ (COSMOlogic, Germany) software (Version C30_1705), which allowed for a predictive screening of their affinity toward target compounds. Furthermore, they were chosen due to their commercial availability, high purity, hydrophobic character, and reported effectiveness in the extraction of aroma compounds from aqueous matrices. The hydrophobic nature of these ILs is critical, as it contributes to minimizing water transport—the primary component in the feed solution—thereby enhancing the overall selectivity of the pervaporation process.

The gelling agent 12-hydroxystearic acid (12-HSA, purity 98%) was also supplied by Merck^®^ (Santiago, Chile) and was used to stabilize the IL phase through gelation. Table 2 summarizes the nomenclature and chemical structure of the selected ILs.

Commercial flat-sheet membranes of polyether block amide (PEBA) and polyoctylmethylsiloxane (POMS) were purchased from Pervatech (Rijssen, The Netherlands). These membranes were selected due to their organophilic characteristics and their suitability for the selective transport of volatile organic compounds in pervaporation processes. In the case of PEBA, the intrinsic balance between hard polyamide (PA) segments and soft polyether (PE) segments governs molecular polarity, rigidity, and transport behavior [18,21,22]. The polyamide blocks introduce polar domains capable of bonding hydrogen, while the polyether segments provide flexible and less polar regions, facilitating the diffusion of organic compounds with different polarities.

### 2.2. Preparation of Asymmetric Membranes Stabilized by Gelation

Prior to gelation, the ILs were dried under vacuum at 82 °C and 1 MPa for 1 h using a thermostatic batch system to remove residual moisture. After drying, the ILs were cooled to room temperature (25 °C), and 1 g of each IL was weighed into glass vials. Subsequently, 12-hydroxystearic acid (12-HSA) was added at concentrations of 0.5%, 1.0%, and 1.5% by weight (Denver scale, model AA-200l, Denver Instrument Company, Arvada, CO, USA), corresponding to the proportion of the gelling agent relative to the IL.

**Figure 1:**
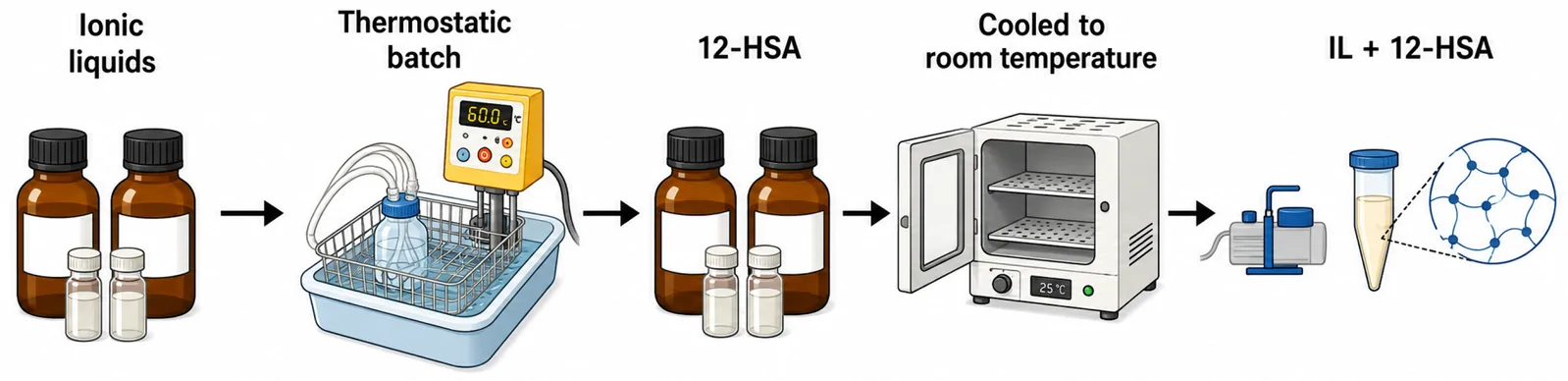
Methodology for the gelation of ILs. The arrows indicate the sequential steps of the ionic-liquid gelation procedure.

The IL and 12-HSA mixtures were heated and homogenized at 80 °C for 4 h using a thermostatic batch system until complete dissolution of the gelling agent was achieved. Gelation was induced by controlled cooling in an oven, decreasing the temperature in steps of −10 °C every 30 min until reaching 20 °C, following the procedure reported in the literature [23]. Successful gel formation was defined operationally by the absence of flow upon vial inversion at room temperature, indicating the formation of a stable three-dimensional network within the ILs liquid phase. The final 12-HAS concentration used for membrane fabrication was selected based on gel stability, absence of macroscopic flow after vial inversion, and visual homogeneity [24]. Figure 1 illustrates the methodology used for the gelation of the ILs.

### 2.3. Fabrication of Trilayer Membranes

**Figure 2:**
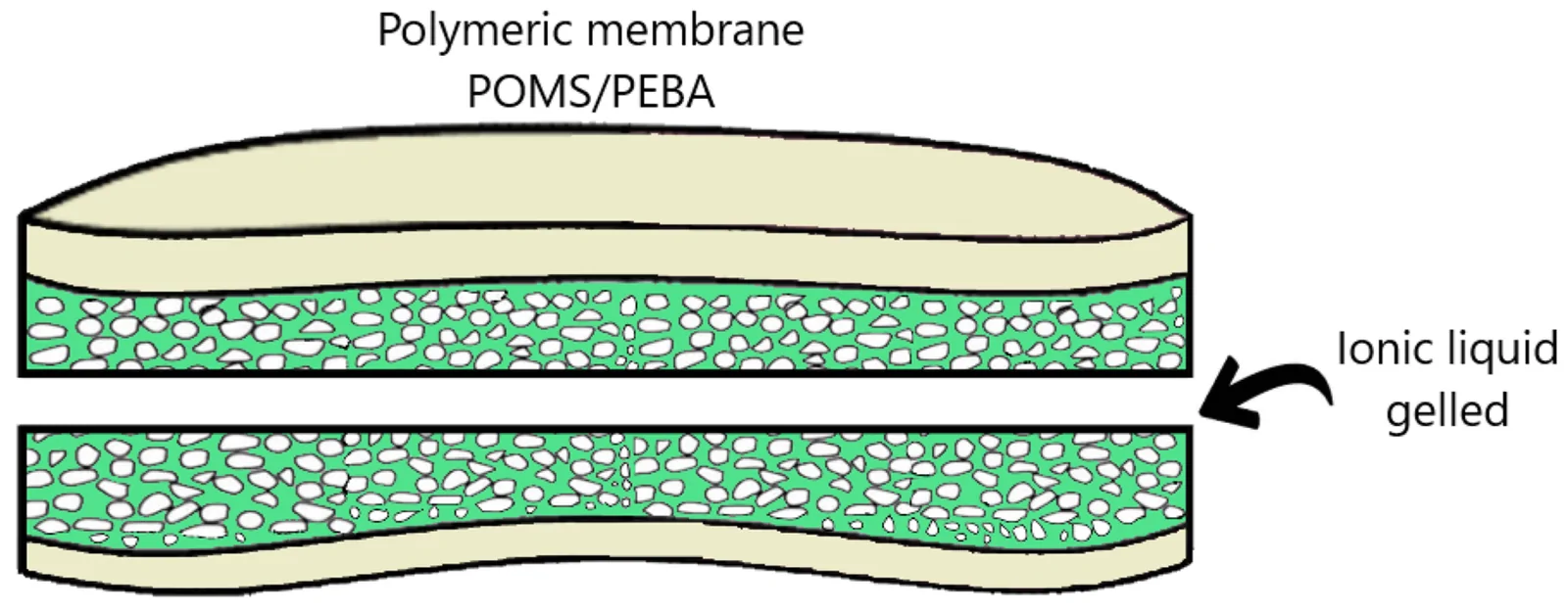
Composite membrane configuration showing the arrangement of the POMS/PEBA matrix with the gelled ionic liquid.

As previously reported by Plaza and Cabezas [12,20], trilayer membranes were fabricated using a polymer/gelled-IL/polymer configuration. Commercial PEBA or POMS membranes were used as the external layers, while the gelled IL constituted the intermediate active layer as shown in Figure 2. Prior to assembly, the polymeric membranes were cut to the required dimensions and conditioned at room temperature.

The trilayer membrane consisted of two commercial composite membranes arranged with their porous non-woven polyester supports facing each other. The gelled ionic liquid was placed between these two porous supports, whereas the dense selective POMS or PEBA layers faced the feed and permeate compartments, respectively. Therefore, the gel was not chemically bonded to the polymer membranes. Instead, it was immobilized by the three-dimensional network formed by 12-HSA and mechanically confined between the two porous supports by compression inside the membrane module. Partial penetration of the gel into the porous support may additionally contribute to its physical retention during operation. The assembled membranes were allowed to equilibrate at room temperature prior to pervaporation experiments [25].

The internal architecture of the trilayer membrane was characterized by cross-sectional scanning electron microscopy (SEM) to assess the penetration of the gelled ionic liquid into the porous membrane support. The SEM images were used to identify morphological changes associated with partial filling of the porous structure and to evaluate the physical retention of the gelled ionic liquid phase within the support, rather than to directly confirm the intact trilayer architecture.

### 2.4. Pervaporation Experiments

**Figure 3:**
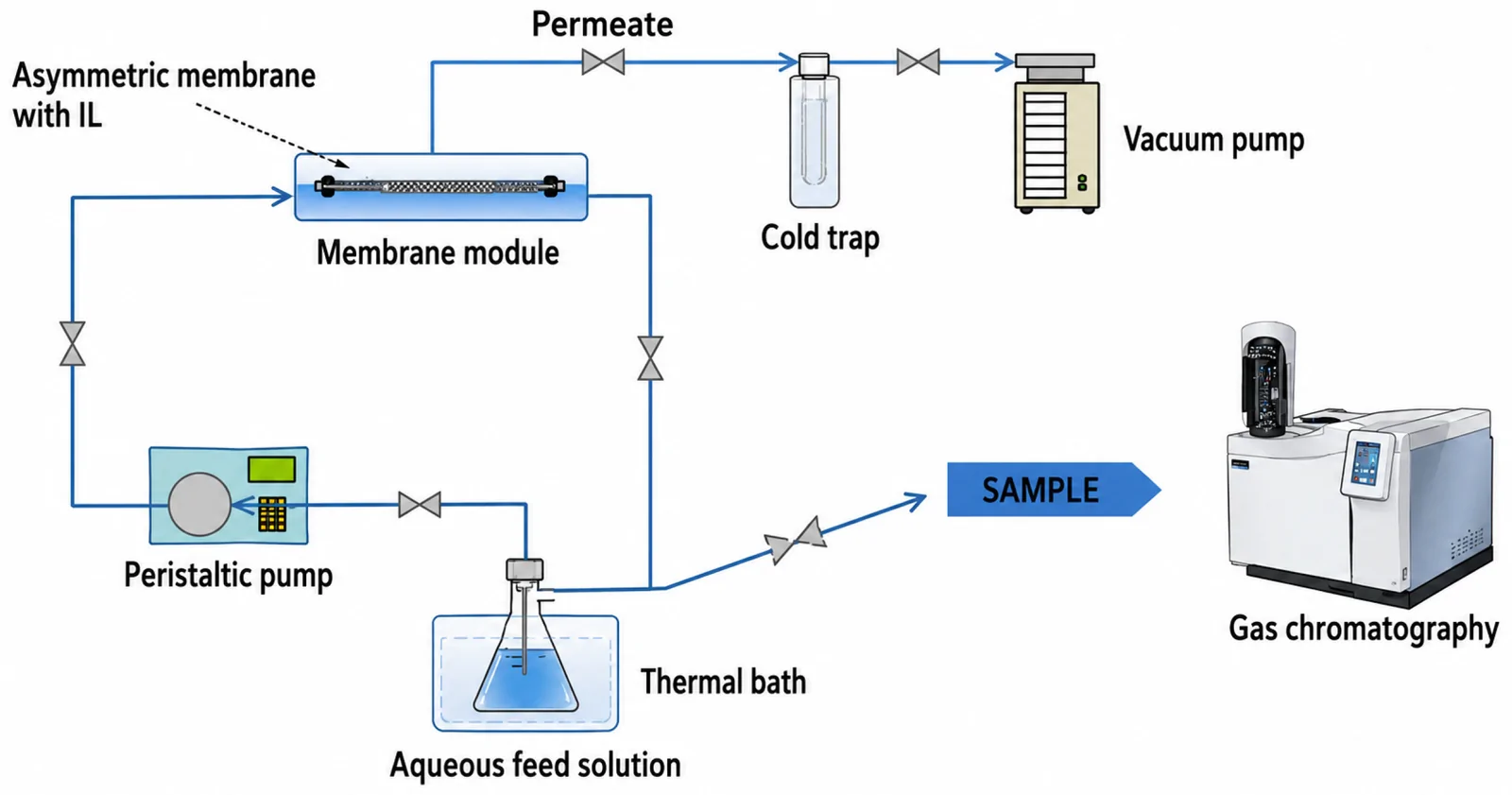
Structural diagram of the pervaporation system.

Pervaporation experiments were conducted using a laboratory-scale setup specifically designed for this application. A schematic representation of the system is shown in Figure 3. The model solution reservoir was connected to a peristaltic pump, enabling continuous recirculation of the feed through a flat-sheet membrane module. The effective membrane area available for mass transfer was 31.36 cm^2^.

On the permeate side, a vacuum pump maintained a constant pressure of 500 mbar, generating the driving force required for selective transport across the membrane. The permeate vapor stream was condensed using a cold trap.

Four trilayer membrane configurations were evaluated, combining two base polymers (PEBA and POMS) and two ILs ([P_1444_][Tf_2_N] and [Bmim][Tf_2_N]). Single-polymer membranes without ILs were used as reference systems. Experiments were carried out at feed flow rates of 100, 500, and 1000 mL min^−1^. Membrane thickness ranged from 0.17 to 0.54 mm, depending on the formulation.

The model AF mixture was prepared by diluting each compound to 1000 ppm in ultrapure water. The feed solution was maintained at 30 °C using a thermostatic bath, while a constant vacuum pressure of 500 mbar was applied on the permeate side. All experiments were performed at least in triplicate, and results are reported as mean values. Table 3 summarizes the experimental parameters and operating conditions used in the pervaporation experiments.

### 2.5. Analytical Methods and Performance Parameters

Prior to the pervaporation experiments, the initial concentrations of the AF compounds in the model mixture were determined by gas chromatography using a Clarus 500 gas chromatograph (PerkinElmer, Shelton, CT, USA), with individual calibration curves and validated analytical methods for each compound. Analyses were performed using an 80/120 Carbopack B column coated with Carbowax 20M (Supelco, Bellefonte, PA, USA) as the stationary phase, helium (Linde Gas Chile S.A., Santiago, Chile) as the carrier gas, and a flame ionization detector (FID) integrated into the Clarus 500 gas chromatograph. Samples were collected at one-hour intervals over a total experimental time of 6 h to evaluate the temporal evolution of compound concentrations.

All pervaporation experiments were conducted in triplicate for each membrane configuration, and the error bars shown in the figures represent the standard deviation of the three replicates.

The transmembrane flux (N) was calculated according to Equation (1):

(1) $$N=-\frac{M\;}{∆t\times A}=\;-\frac{∆C\times \;10^{-6}\times V}{∆t\times A}$$

where M is the mass transferred (kg), ∆t the time interval from $t_{1}$ to $t_{2}$ (h), A the effective membrane area (m^2^), ∆C the concentration difference between $t_{1}$ and $t_{2}$ (mg L^−1^), and V the volume of the solution used (L). This concept is crucial, as it quantifies the efficiency of water and compound transport through the membrane [12]. A higher flux density indicates better performance of the pervaporation system.

Additionally, the extraction percentage for each compound was calculated using the following equation:

(2) $$Extraction\;\%=\frac{C_{0}-C_{t}}{C_{0}}\;*\;100$$

where $C_{0}$ is the initial compound concentration (ppm) and $C_{t}$ the concentration at the evaluated time point (ppm). These metrics allow for comparison of the efficiency of each membrane system under different operating conditions.

To evaluate the efficiency of the membranes in separating aroma and fragrance compounds from the aqueous phase, the selectivity toward water was calculated for each compound i [26]. The separation factor is defined as:

(3)  βi/w={Ci−pCw−pCi−fCw−f}

where $C_{i-p}$ and $C_{i-f}$ are the concentrations of compound i in the total mass of the collected permeate and in the feed solution after the pervaporation process, respectively. $C_{w-p}$ and $C_{w-f}$ correspond to the water concentrations in the permeate and feed solution, respectively. This dimensionless parameter reflects the membrane’s preferential transport of organic compounds relative to water.

In addition, the enrichment factor (E) was calculated to quantify the extent to which each compound is concentrated in the permeate relative to the feed solution [24]. This parameter is defined as:

(4) $$E=\left\{\frac{C_{i-p}}{C_{i-f}}\right\}$$

### 2.6. Mass Transfer Analysis

The mass transfer of water and AFs through the evaluated membranes was analyzed using a resistance-in-series approach considering contributions from both the liquid boundary layer and the membrane [27,28,29,30]. The analysis was applied to the support membranes (POMS and PEBA) and to the ionic-liquid-based sandwich membranes POMS/[P_1444_][Tf_2_N]/POMS, POMS/[Bmim][Tf_2_N]/POMS, PEBA/[P_1444_][Tf_2_N]/PEBA, PEBA/[Bmim][Tf_2_N]/PEBA. Experimental transmembrane fluxes of water and AFs were obtained from pervaporation experiments and used to calculate the overall mass transfer coefficient.

The overall mass transfer coefficient $K_{overall,i}$ was determined from the measured flux $N_{i}$ according to:

(5) $$N_{i}=K_{overall,i}\;\gamma_{i}\;x_{i}\;P_{i}^{sat}$$

where $x_{i}$ is the mole fraction of compound i in the feed, $\gamma_{i}$ is its activity coefficient in the liquid phase, and $P_{i}^{sat}$ is the saturation vapor pressure at 30 °C. For water, γ=1 and x=1, yielding:

(6) $$N_{w}=K_{overall,w}P_{w}^{sat}$$

The feed composition was assumed to be 1000 ppm of each AF in water. Vapor pressures were obtained from literature data, while activity coefficients at infinite dilution were estimated using the NRTL model.

The convective mass transfer coefficient in the liquid phase ($k_{L}$) was estimated from hydrodynamic correlations for a rectangular channel. The module channel had dimensions of 5.6 cm × 5.6 cm with a height of 3 mm and a feed flow rate of 1000 mL·min^−1^. Reynolds, Schmidt and Sherwood numbers were calculated as:

(7) $$Re=\frac{\rho uD_{eq}}{\mu }$$

(8) $$Sc=\frac{\mu }{\rho D_{i}}$$

(9) $$Sh=4.86+\frac{0.32(4X_{m}{)}^{-1.2}}{1+0.24(4X_{m}{)}^{-0.7}Sc^{0.17}}$$

where $D_{i}$ is the diffusion coefficient of compound i in water and $D_{eq}$ is the hydraulic diameter of the channel. The liquid-phase mass transfer coefficient was then obtained from:

(10) $$k_{L}=\frac{Sh\;D_{i}}{D_{eq}}$$

Assuming a resistance-in-series model, the overall mass transfer coefficient can be expressed as:

(11) $$\frac{1}{K_{overall,i}}=\frac{1}{K_{L,i}}+\frac{1}{K_{M,i}}$$

where $K_{L,i}$ and $K_{M,i}$ correspond to the liquid-phase and membrane mass transfer coefficients, respectively. The relative contributions of each resistance were calculated as:

(12) $$R_{L}(\%)=\frac{1/K_{L,i}}{1/K_{overall,i}}\times 100$$

(13) $$R_{M}(\%)=\frac{1/K_{M,i}}{1/K_{overall,i}}\times 100$$

Membrane permeability for each compound was obtained from:

(14) $$P_{i}=K_{M,i}\;\delta$$

where δ represents the membrane thickness.

Finally, membrane selectivity relative to water was calculated as:

(15) $$\alpha_{i,w}=\frac{P_{i}}{P_{w}}$$

In the present study, the pervaporation separation index (PSI) was calculated using the permeability-based membrane selectivity ($\alpha_{i,w}$) rather than the experimental separation factor (${\;\beta }_{i/w}$). This formulation has also been reported in the pervaporation literature and was selected here because it combines the total permeation flux with the intrinsic membrane selectivity derived from permeability values, thereby allowing membrane performance to be evaluated while accounting for the thermodynamic driving force [17]. Accordingly, the PSI was determined as:

(16) $$PSI=J_{t}(\alpha_{i,w}-1)$$

where $J_{t}$ is the transmembrane total flux. These parameters allowed comparison of membrane performance in terms of permeability, selectivity, and overall separation efficiency.

## 3. Results and Discussions

### 3.1. Gelation and Morphological Characterization of Asymmetric Trilayer Membranes

**Figure 4:**
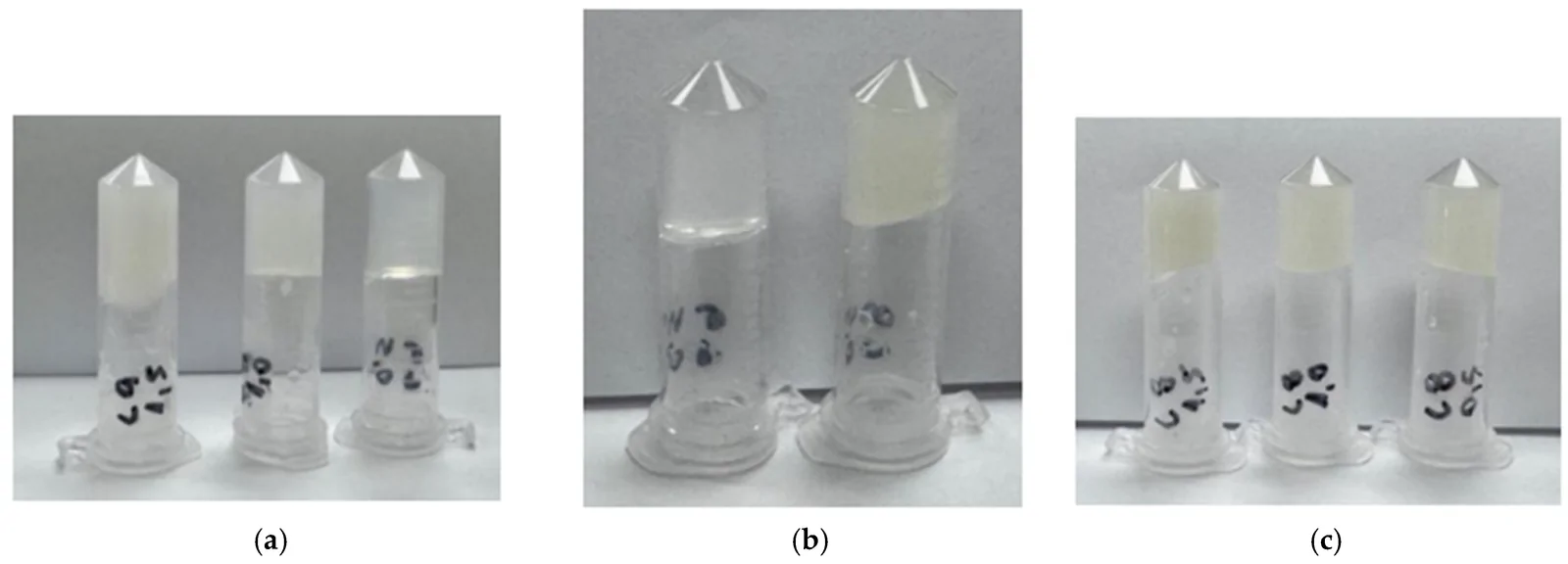
Gelled ILs at all tested concentrations of 12-HAS. (**a**) Concentrations [P_1444_][Tf_2_N]. (**b**) 0.5% of each IL. (**c**) Concentrations [Bmim][Tf_2_N].

Asymmetric trilayer membranes were prepared by gelation of the ILs [P_1444_][Tf_2_N] and [Bmim][Tf_2_N] using 12-hydroxystearic acid (12-HSA) at concentrations of 0.5%, 1.0%, and 1.5% *w*/*w*. Figure 4a–c show that all formulations produced self-supporting gels that retained their shape under inversion for at least 10 min, confirming successful immobilization of the IL phase.

A clear difference in optical appearance was observed between the two gelled ILs (Figure 4b). The [P_1444_][Tf_2_N] gels exhibited a white-transparent aspect, whereas the [Bmim][Tf_2_N] gels showed a yellowish coloration. This behavior is attributed to specific interactions between 12-HSA and the different IL cations, which affect the optical properties of the gel network [13,31].

The lowest gelator concentration (0.5% *w*/*w*) was selected as optimal, as it ensured structural stability while minimizing internal mass-transfer resistance and maximizing the effective IL content available for selective transport [32]. Based on this formulation, trilayer membranes were assembled using 0.48 g of gelled [P_1444_][Tf_2_N] and 0.52 g of gelled [Bmim][Tf_2_N], corresponding to an IL-layer thickness of ~100 µm. The membranes were assembled with the porous polyester support facing the gel, while the dense selective polymer layers faced outward.

**Figure 5:**
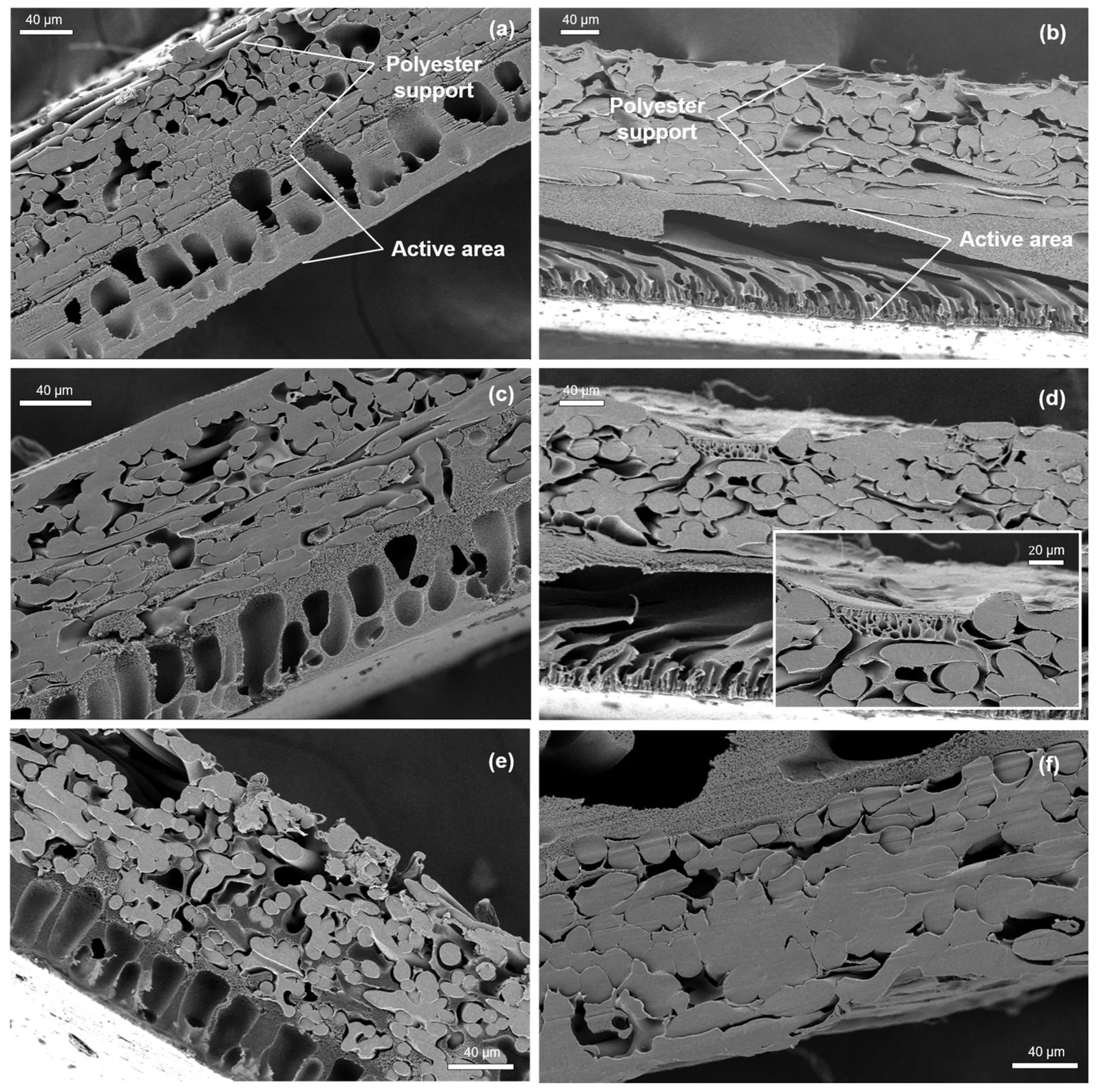
Cross-sectional SEM images of (**a**) POMS, (**b**) PEBA, (**c**) POMS/[Bmim][Tf_2_N], (**d**) PEBA/[Bmim][T_f2_N], (**e**) POMS/[P_1444_][Tf_2_N], and (**f**) PEBA/[P_1444_][Tf_2_N]. The inset in panel (**d**) shows a higher-magnification view of the porous support/gel region.

Figure 5 compares the cross-sectional morphology of the neat POMS and PEBA composite membranes with that of membranes recovered after assembly with the gelled ionic liquid intermediate phase. During operation, the gel was physically confined between two composite membranes by mechanical compression within the membrane holder, with the porous polyester supports facing the gel and the dense selective layers facing outward. This configuration provided intimate contact between the gel and the porous supports and likely promoted partial interpenetration of the gel into the interconnected voids of the support structure. Consistent with this interpretation, the SEM images of the membranes exposed to the gel show regions with partially filled interfiber voids and a more compact appearance compared with the neat membranes. However, these morphological features should be regarded as indirect evidence of gel interpenetration rather than direct identification of the ionic liquid. Upon opening the membrane holder, the mechanical compression maintaining the trilayer assembly was released, causing the layers to separate and part of the non-confined gel to be lost during SEM sample preparation. Therefore, the SEM images do not reproduce the intact trilayer architecture under operating conditions but provide morphological evidence consistent with partial retention of the gel within the porous support.

The lowest gelator concentration (0.5% *w*/*w*) was selected as optimal, as it ensured structural stability while minimizing internal mass-transfer resistance and maximizing the effective IL content available for selective transport [32]. Based on this formulation, trilayer membranes were assembled using 0.48 g of gelled [P_1444_][Tf_2_N] and 0.52 g of gelled [Bmim][Tf_2_N], corresponding to an IL-layer thickness of ~100 µm.

### 3.2. Determination of Extraction Capacities

The extraction performance of the reference single-layer membranes was first evaluated to establish a baseline for comparison with trilayer systems incorporating gelled ILs. POMS membranes are highly hydrophobic due to their siloxane-based structure, which favors the permeation of nonpolar and weakly polar compounds such as aldehydes and long-chain alcohols [33]. In contrast, PEBA consists of alternating polyamide (PA) and polyether (PE) blocks, whose relative proportion governs the polarity, rigidity, and transport behavior of the membrane through microphase-separated domains that modulate solubility and diffusivity [34].

**Figure 6:**
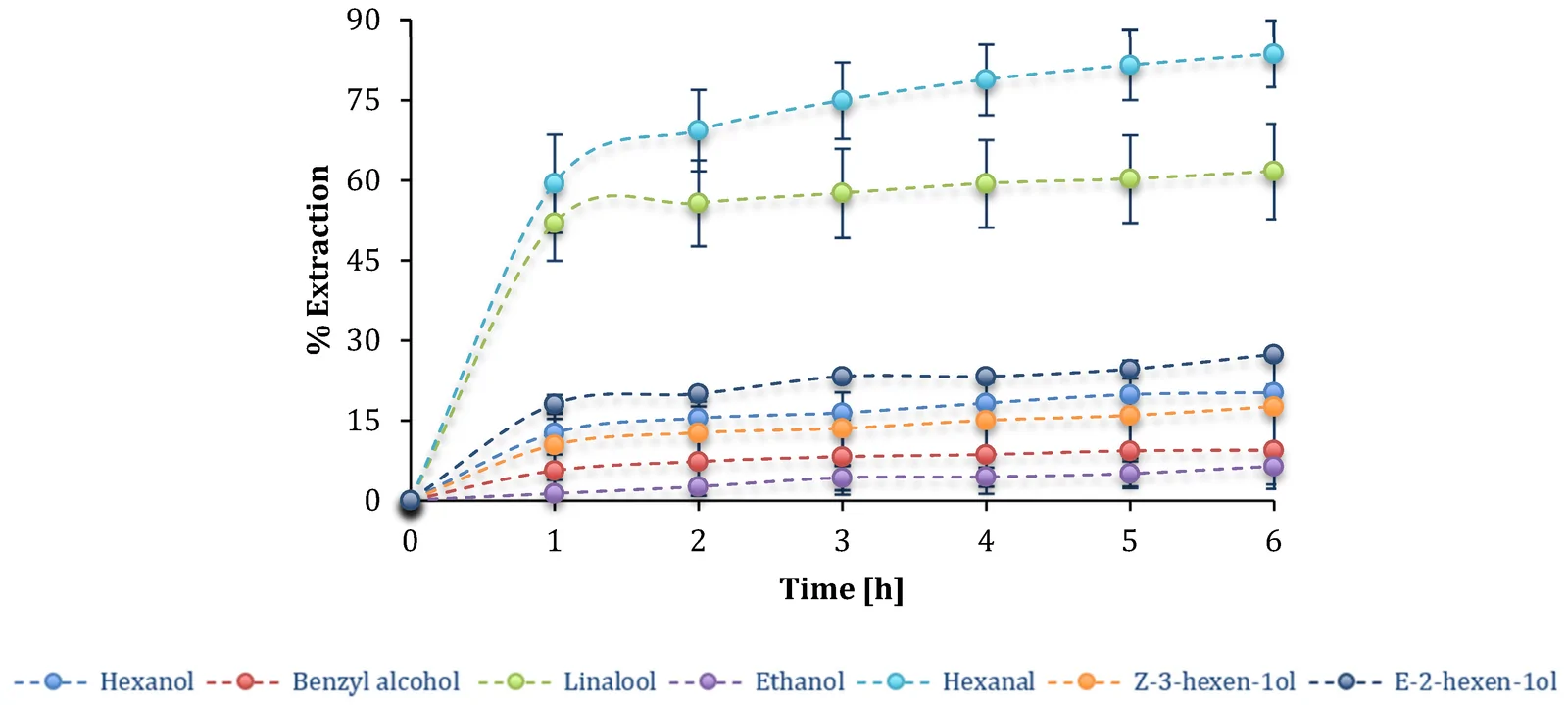
Extraction percentage of compounds using the POMS membrane at a flow rate of 1000 (mL min^−1^).

**Figure 7:**
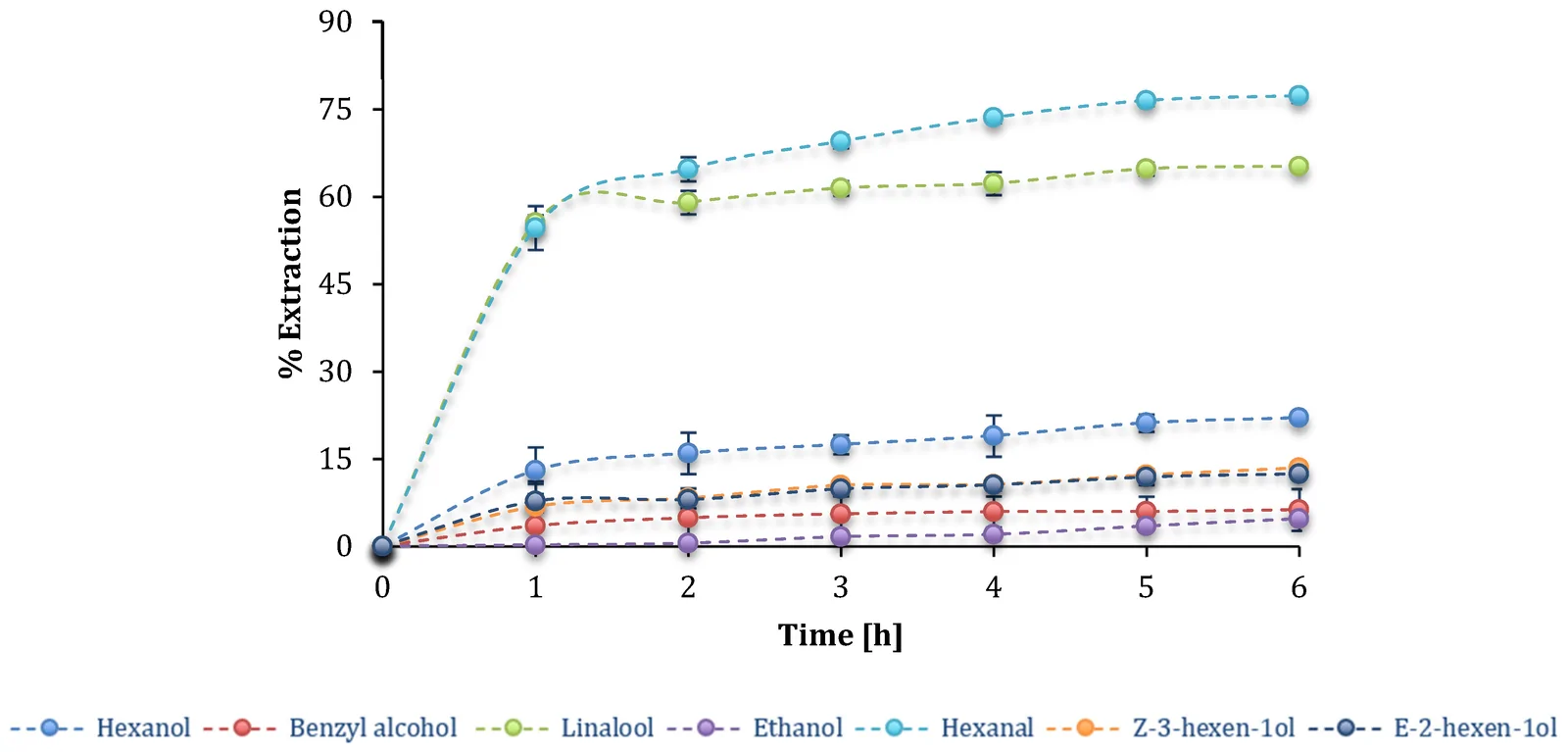
Extraction percentage of compounds using the PEBA membrane at a flow rate of 1000 (mL min^−1^).

Figure 6 and Figure 7 show the time-dependent extraction percentage for single-layer POMS and PEBA membranes, respectively, at a feed flow rate of 1000 mL min^−1^.

As shown in Figure 6 and Figure 7, both membranes exhibited higher extraction of hexanal, and linalool compared with the other compounds. This behavior is associated with their relatively low polarity, high affinity for organophilic phases, and favorable diffusion through polymer matrices with limited hydrogen-bonding capacity [35,36]. In POMS, hexanal extraction was approximately 20% higher than that of linalool, reflecting the stronger affinity of this hydrophobic siloxane matrix for less polar aldehydes. In PEBA, this difference was reduced to approximately 10%, consistent with its higher polarity and enhanced interaction with oxygenated compounds.

Differences in extraction behavior between the membranes can be directly related to their polymer structure. POMS, characterized by low surface energy and weak specific interactions, preferentially facilitates the transport of nonpolar species. In contrast, the presence of polar polyamide and polyether domains in PEBA enables dipole–dipole and hydrogen-bond interactions with moderately polar molecules.

The different behavior between membranes can be rationalized based on their polymer structure. POMS is a highly hydrophobic siloxane-based polymer with low surface energy and limited ability to form specific interactions, favoring the transport of nonpolar or weakly polar compounds. In contrast, PEBA contains polyether and polyamide segments, which increase its polarity and enable dipole–dipole and hydrogen-bond interactions with moderately polar molecules.

Accordingly, hexanol exhibited higher extractions in PEBA than in POMS, with recovery values of approximately 22% and 12%, respectively. This trend is attributed to the moderate polarity and conformational flexibility of hexanol, which enhances its interaction with the polar domains of PEBA. Conversely, the hexanol isomers showed lower extraction levels in both membranes, likely due to their more rigid molecular structures and reduced affinity for the polymer matrices.

In POMS, E-2-hexen-1-ol was extracted slightly more efficiently than hexanol, consistent with its lower polarity and improved compatibility with the hydrophobic siloxane matrix phase [37]. In PEBA, E-2-hexen-1-ol and Z-3-hexen-1-ol exhibited similar extraction levels, both lower than hexanol, in agreement with their comparable polarity and reduced conformational flexibility [38].

The extraction performance of the trilayer membranes incorporating gelled ILs was evaluated under three feed flow rates (100, 500, and 1000 mL min^−1^). The results are presented sequentially for the POMS/[P_1444_][Tf_2_N]/POMS, POMS/[Bmim][Tf_2_N]/POMS, PEBA/[P_1444_][Tf_2_N]/PEBA and finally PEBA/[Bmim][Tf_2_N]/PEBA configurations.

**Figure 8:**
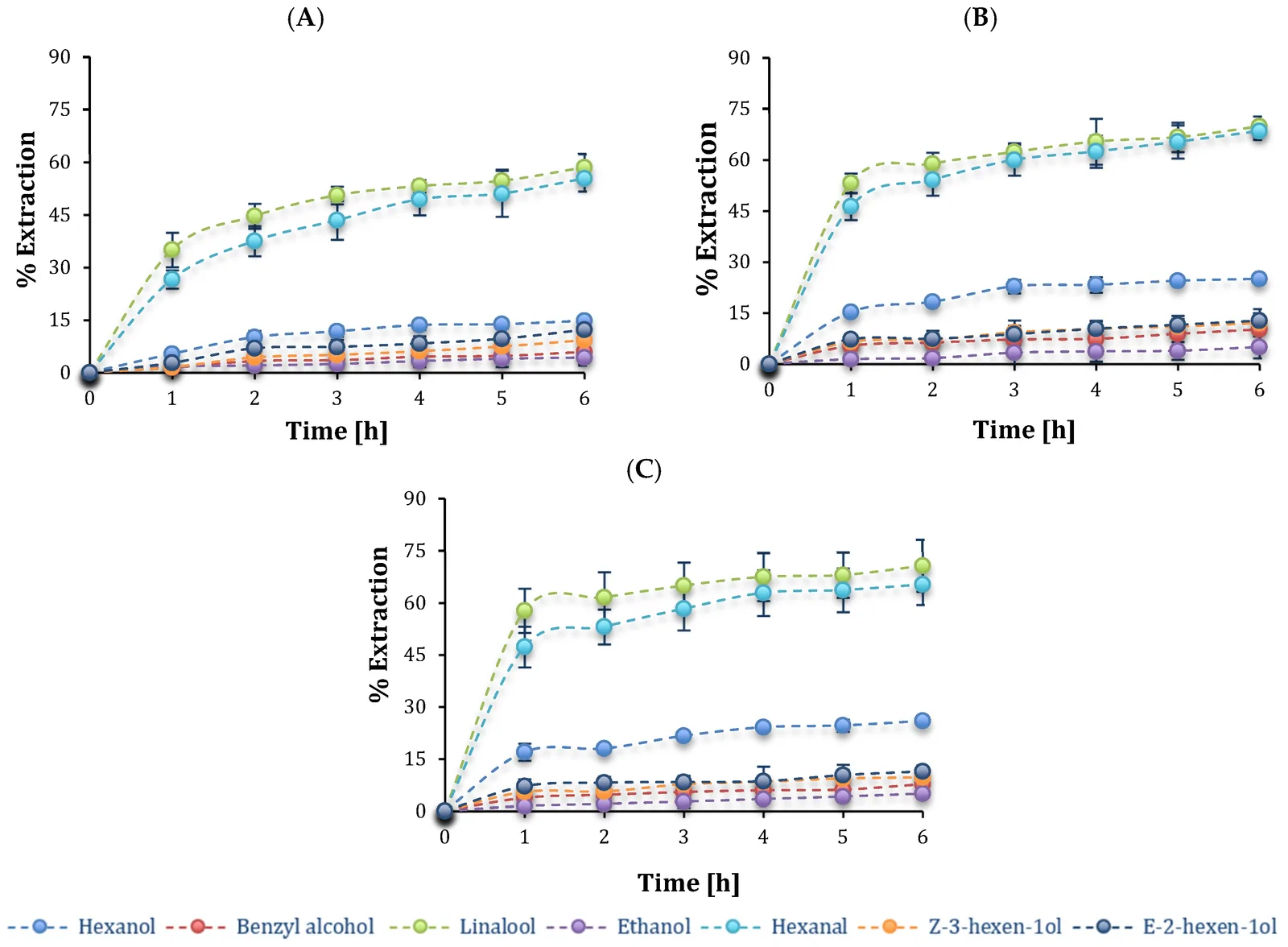
Extraction percentage using the POMS/[P_1444_][Tf_2_N]/POMS membrane at flow rates (**A**) 100, (**B**) 500, and (**C**) 1000 (mL min^−1^).

As shown in Figure 8, increasing the feed flow rate markedly enhanced extraction performance for all compounds in the POMS/[P_1444_][Tf_2_N]/POMS membrane. This effect is associated with reduced external mass transfer resistance under higher hydrodynamic conditions. Linalool consistently exhibited slightly higher extraction than hexanal, attributed to its hydroxyl group, which enables hydrogen-bond interactions with the IL, increasing solubility and retention within the gel phase [39]. Although hexanal is more volatile, its weaker interaction with the IL limits its relative extraction [40].

**Figure 9:**
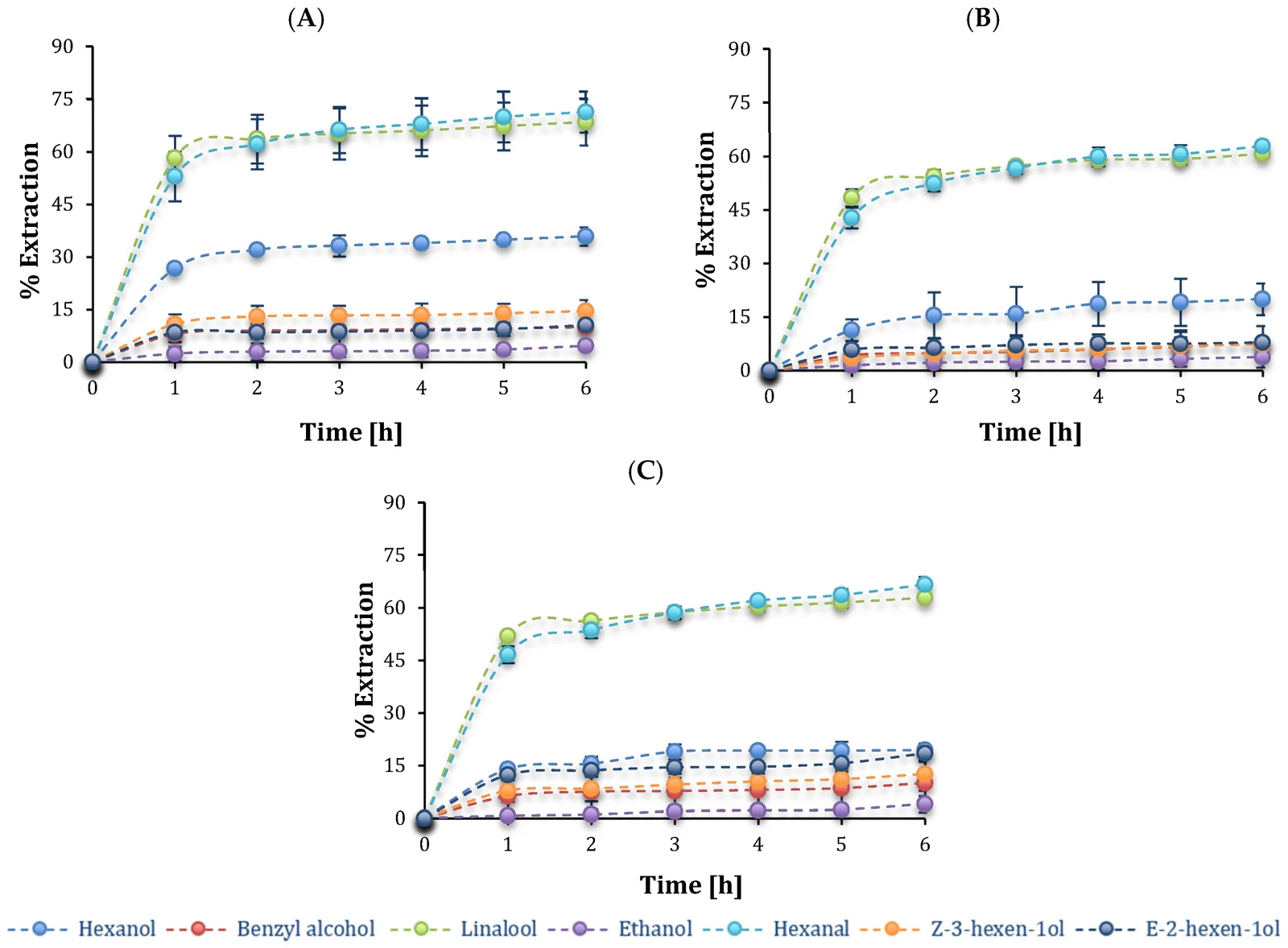
Extraction percentage using the POMS/[Bmim][Tf_2_N]/POMS membrane at flow rates (**A**) 100, (**B**) 500, and (**C**) 1000 (mL min^−1^).

In the POMS/[Bmim][Tf_2_N]/POMS system (Figure 9), a crossover between linalool and hexanal extraction was observed after approximately 3 h of operation. Initially, linalool exhibited higher extraction due to its greater solubility in the polymer matrix; however, at longer times and higher flow rates, hexanal became dominant owing to its higher volatility and weaker interaction with the IL. Hexanol extraction decreased at higher flow rates, likely due to retention within the IL phase, while the hexenol isomers displayed flow-dependent behavior. At low flow rates, Z-3-hexen-1-ol was preferentially extracted, whereas at higher flow rates E-2-hexen-1-ol dominated, reflecting reduced interaction-based retention when liquid-phase mass transfer became controlling [32,41].

**Figure 10:**
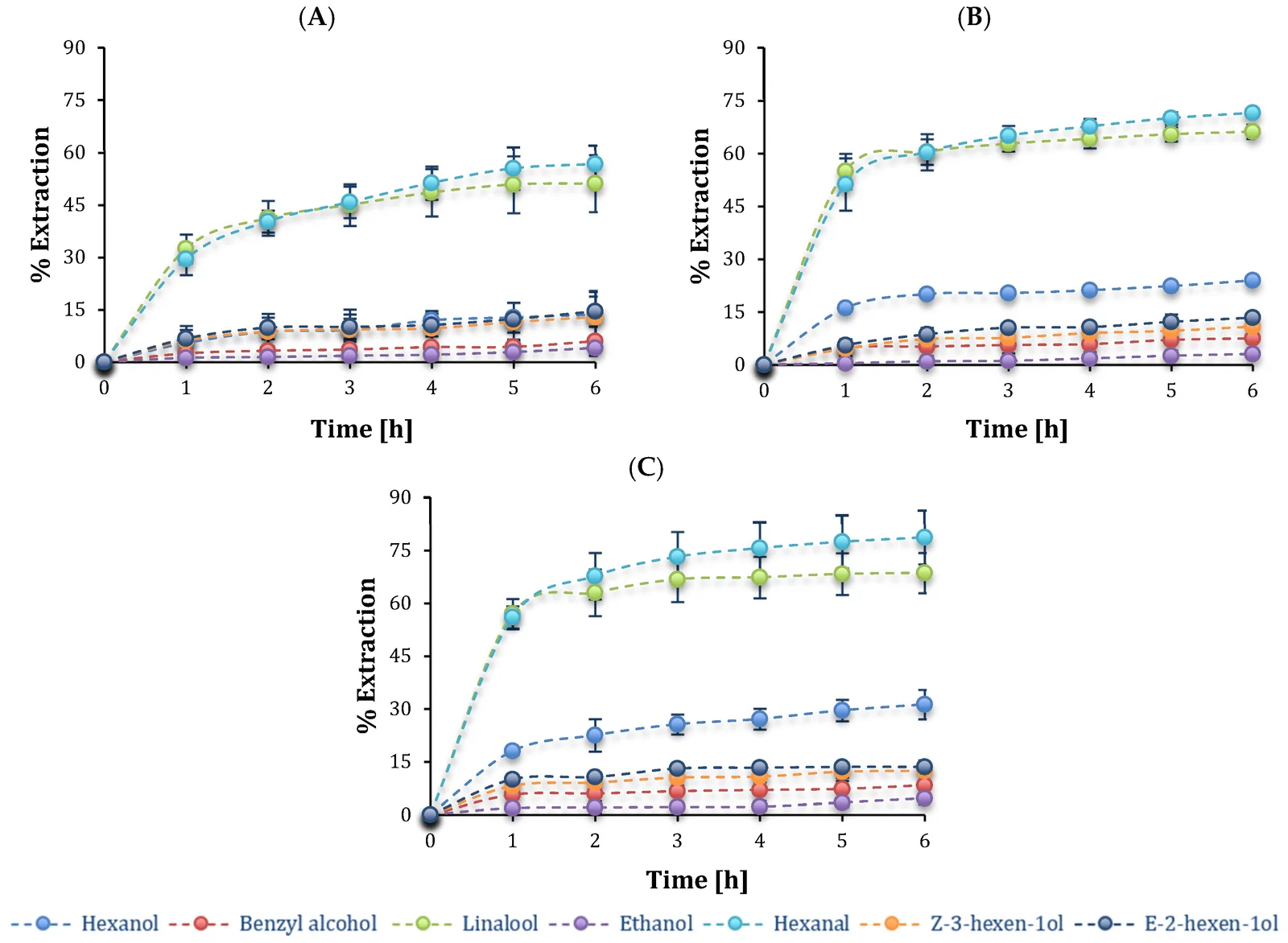
Extraction percentage using the PEBA/[P_1444_][Tf_2_N]/PEBA membrane at flow rates (**A**) 100, (**B**) 500, and (**C**) 1000 (mL min^−1^).

For the PEBA/[P_1444_][Tf_2_N]/PEBA membrane (Figure 10), similar extraction levels for hexanol, and the hexenol isomers were observed at low flow rates, indicating diffusion-controlled transport. At higher flow rates 80%, driven by its lower molecular size and higher volatility. Hexanol extraction also increased with flow rate, reflecting its high solubility in the membrane and the enhanced hydrophobic character imparted [42,43,44,45]. In contrast, Z-3-hexen-1-ol, showed reduced extraction due to its lower affinity for the IL phase [46,47].

**Figure 11:**
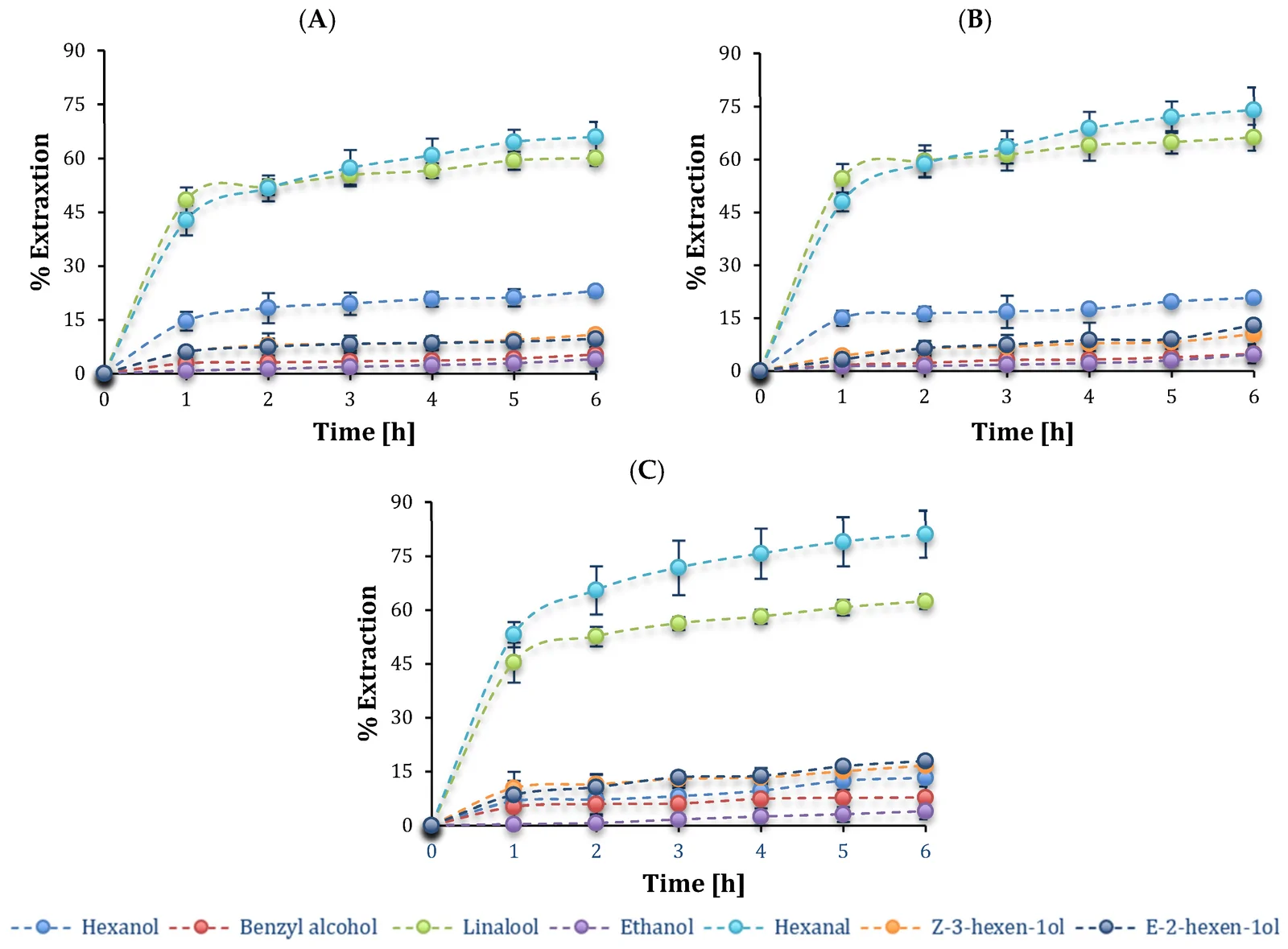
Extraction percentage using the PEBA/[Bmim][Tf_2_N]/PEBA membrane at flow rates (**A**) 100, (**B**) 500, and (**C**) 1000 (mL min^−1^).

In the PEBA/[Bmim][Tf_2_N]/PEBA membrane (Figure 11), crossover points between hexanal and linalool extraction occurred at approximately 2 h, independent of flow rate. Although overall extraction increases with flow, the relative difference between both compounds remained constant, with hexanal exceeding 80% extraction. Linalool showed higher initial extraction due to its solubility in PEBA [48], but its interaction with [Bmim][Tf_2_N], including π–π and van der Waals interactions, progressively limits its mobility over time [49]. Hexanol extraction decreases with an increasing flow rate, while E-2-hexen-1-ol and Z-3-hexen-1-ol showed improved extraction at higher flows, reaching approximately 20%.

Across all configurations, hexanol and linalool were consistently among the most efficiently extracted compounds. Statistical analysis (two-way ANOVA with replication) confirmed that feed flow rate significantly affected extraction only in systems containing [P_1444_][Tf_2_N] (*p* < 0.05), whereas no significant flow-rate effect was detected for [Bmim][Tf_2_N] based membranes (*p* > 0.09). In all cases, the dominant factor was the nature of the compound, with no significant interaction between flow rate and compound type. These differences are attributed to the higher hydrophobic of [P_1444_][Tf_2_N], which favors hexanol transport, whereas the more polar [Bmim][Tf_2_N] promotes retention and mobility limitations [50,51].

Finally, ethanol and benzyl alcohol showed the lowest extraction in all systems. Ethanol never exceeded 5% extraction due to its high affinity for water and low vapor pressure [52]. Benzyl alcohol exhibited slightly higher extraction in POMS-based membranes, and marginal improvement in [Bmim][Tf_2_N] systems due to π–π interactions; however, diffusion limitations restricted its overall transport, particularly in [P_1444_][Tf_2_N]-based membranes [53,54].

### 3.3. Transmembrane Flux of Aroma Compounds

**Figure 12:**
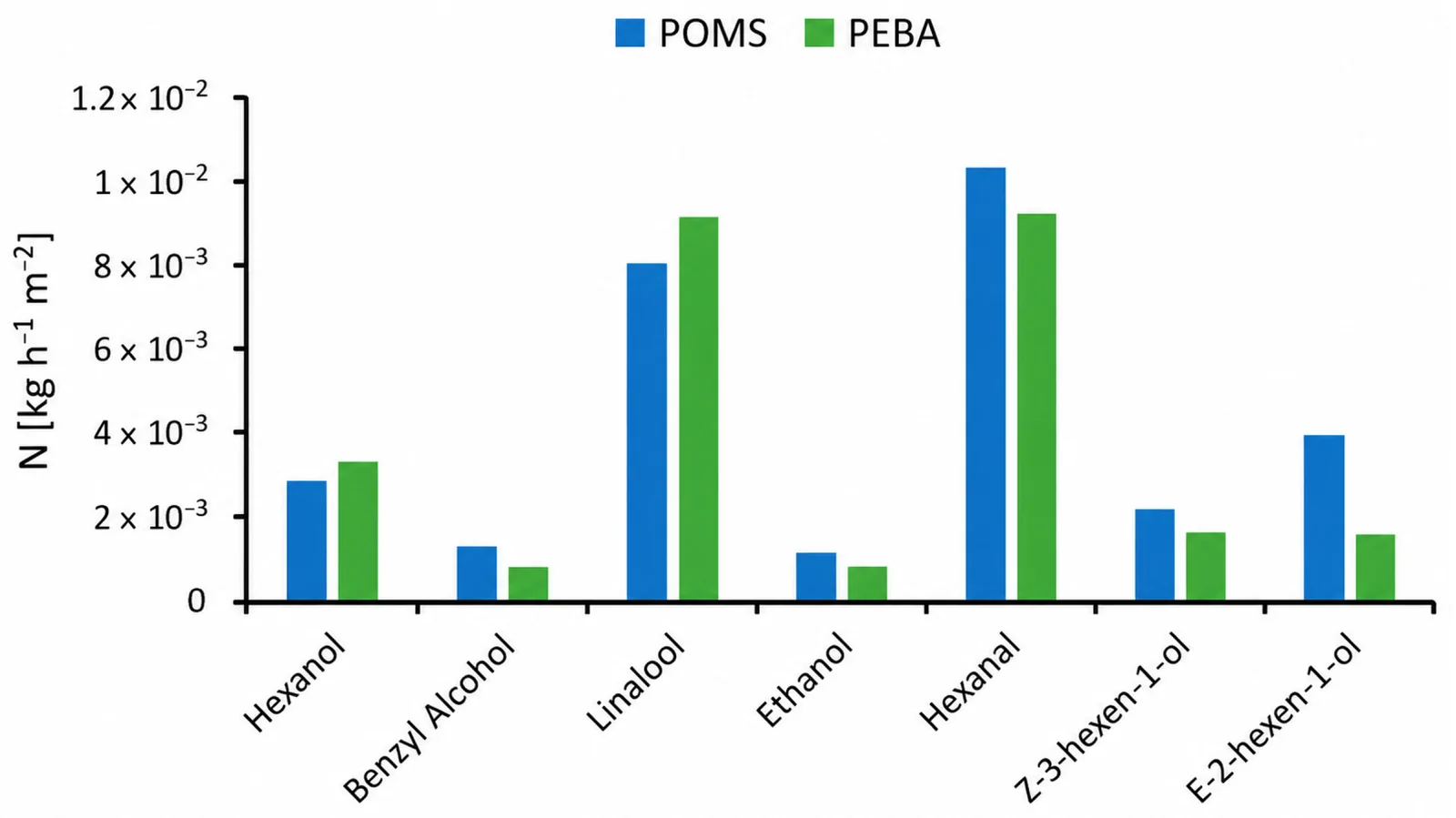
Total Transmembrane flux $N_{x}$ (kg h^−1^ m^−2^) of the compounds through POMS and PEBA membranes at 1000 mL min^−1^.

The transmembrane flux $\left(N_{i}\right)$ of the AF compounds was determined from the mass balance in the feed phase, considering the change in solute concentration between two consecutive time intervals. As shown in Figure 12, the membranes with POMS support exhibited higher flux densities for all evaluated compounds compared with the PEBA membranes, indicating greater transport efficiency. For example, the hexanal flux in POMS was approximately 12% higher than in PEBA, which is consistent with the better compatibility of POMS with short-chain aldehydes [55].

Figure 12 further shows that hexanal and linalool exhibited the highest transmembrane fluxes among all analyzed compounds, reaching values clearly above those of the remaining analytes. This result confirms that both compounds dominate mass transport through the membranes, whereas hexanol, the hexanol isomers, benzyl alcohol, and especially ethanol showed considerably lower flux values, indicating a reduced contribution to the overall permeation process.

Within this context, differences between the hexanol isomers were attributed to molecular conformation effects, which influence diffusivity through polymeric membranes, resulting in higher mobility in POMS compared with PEBA. This behavior is consistent with the literature reports describing the structural influence of unsaturation and chain geometry on diffusion through polymeric matrices [56]. In contrast, benzyl alcohol transport was favored in POMS, while linalool and hexanal showed relatively higher permeability in the PEBA membrane. This behavior can be associated with the higher polarity of these compounds and the presence of polyamide domains in the PEBA matrix, which promotes solubility and diffusion. Finally, ethanol exhibited very low flux values and no significant differences between membranes, in agreement with previous reports describing its limited permeability in hydrophobic membrane systems [57].

In summary, although variations in selectivity between POMS and PEBA membranes were observed, the permeation process was clearly dominated by the permeation of hexanal and linalool, whose high mobility determined the overall transmembrane flux behavior relative to the other aroma compounds.

**Figure 13:**
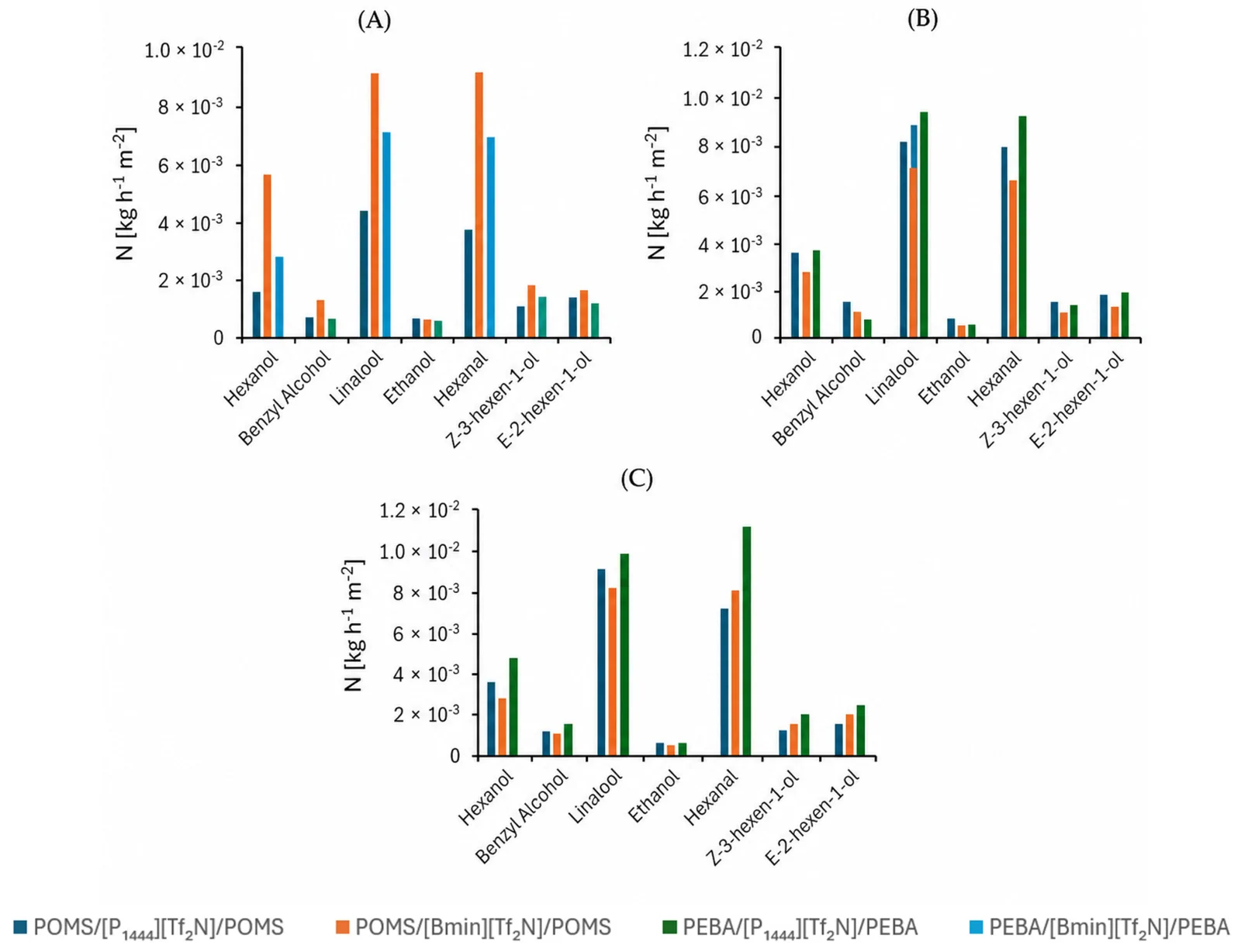
Transmembrane flux densities $N_{x}$ (kg h^−1^ m^−2^) of the compounds through POMS/[P_1444_][Tf_2_N]/POMS, POMS/[Bmim][Tf_2_N]/POMS, PEBA/[P_1444_][Tf_2_N]/PEBA, and PEBA/[Bmim][Tf_2_N]/PEBA membranes at flow rates of (**A**) 100, (**B**) 500, and (**C**) 1000 mL min^−1^.

The transmembrane flux densities of the aroma compounds through the trilayer membranes are presented in Figure 13 for feed flow rates of (A) 100, (B) 500, and (C) 1000 mL min^−1^. The analysis reveals distinct transport behaviors depending on both the compound and the membrane configuration.

As shown in Figure 13, hexanol exhibited higher fluxes in the POMS/[Bmim][Tf_2_N]/POMS membranes at low flow rates, which can be attributed to favorable interactions between the hydrophobic POMS matrix and the imidazolium-based IL [58,59]. Benzyl alcohol showed low permeability in all membrane configurations, with only a minor increase observed in POMS/[Bmim][Tf_2_N]/POMS, suggesting a combined effect of polymer matrix properties and IL affinity on solubility and diffusion.

Linalool displayed consistently high flux values across all trilayer membranes, reaching a maximum value in PEBA/[Bmim][Tf_2_N]/PEBA. This behavior can be explained by the flexibility of the PEBA matrix and the high solubility of linalool in imidazolium-based ILs, which together enhanced mass transport. In contrast, POMS/[P_1444_][Tf_2_N]/POMS exhibited comparatively lower linalool fluxes, confirming the weaker interaction between phosphonium-based ILs and terpenoid compounds. Ethanol presented the lowest flux values in all configurations, with minimal variation as a function of flow rate.

Hexanal exhibited the highest transmembrane fluxes in all membrane configurations and flow conditions, as clearly shown in Figure 13, reaching maximum values in PEBA/[Bmim][Tf_2_N]/PEBA membranes. This behavior is attributed to the combined effect of polar segments in the PEBA matrix and the strong affinity of aldehyde groups for the imidazolium IL. The hexenol isomers (Z-3-hexen-1-ol and E-2-hexen-1-ol) also permeated more efficiently in membranes containing [Bmim][Tf_2_N], particularly in PEBA-based systems, confirming that the combination of a flexible polymer matrix and an imidazolium IL enhance mass transport performance [11].

Overall, Figure 13 demonstrates that transmembrane transport through the trilayer membranes is predominantly governed by hexanal and linalool, whose fluxes largely exceed those of the remaining compounds. Although variations in flux were observed depending on the polymeric support and IL employed, the global transport behavior of the system was mainly defined by these two compounds due to their higher mobility and favorable interactions within the membrane structure [35,37].

As shown in Table 4, the PEBA-support membrane exhibited the highest water flux (0.312 kg h^−1^ m^−2^). This behavior is associated with the presence of polar polyamide segments within the PEBA structure, which facilitate water sorption and diffusion through specific polar interactions, rather than indicating a fully hydrophilic character of the membrane.

In contrast, the POMS-support membrane showed significantly lower water flux (0.069 kg h^−1^ m^−2^), which is consistent with its hydrophobic siloxane backbone, low surface energy, and limited water uptake and transport through the polymer matrix [36].

The incorporation of ILs significantly reduced water permeability in all trilayer configurations, with fluxes ranging from 0.006 to 0.039 kg h^−1^ m^−2^. This reduction is attributed to the formation of an additional diffusive barrier introduced by the IL layer, which decreases water solubility and mobility within the membrane structure [28,51]. Among the modified membranes, PEBA/[Bmim][Tf_2_N]/PEBA exhibited the lowest water flux (0.013 kg h^−1^ m^−2^), indicating enhanced organic/water selectivity, whereas POMS/[Bmim][Tf_2_N]/POMS showed the highest value (0.039 kg h^−1^ m^−2^), consistent with the higher flexibility and hydrophobicity of POMS [33].

Membranes containing [P_1444_][Tf_2_N] consistently showed lower water fluxes than those containing [Bmim][Tf_2_N], as the phosphonium cation increases hydrophobicity and restricts water diffusion through the IL interlayer [41]. Longer operation times (12–22 h) also led to progressive flux stabilization, particularly in POMS-based configurations, suggesting the establishment of a dynamic equilibrium between sorption and diffusion within the IL phase [23,60].

Overall, these results confirm that both polymer hydrophobicity and IL chemistry are key factors governing water transport. The PEBA/[Bmim][Tf_2_N]/PEBA membrane proved to be the most effective configuration for minimizing water permeation while maintaining selective recovery of aroma compounds [59,61].

The enrichment factor (E) results presented in Table 5 show that the membranes modified with ILs achieved substantially higher concentrations of aroma compounds relative to water compared with the unmodified membranes. In particular, PEBA/[P_1444_][Tf_2_N]/PEBA exhibited the highest E values for hexanal (≈1625.9) and linalool (≈962.6), indicating excellent enrichment capability.

Similarly, POMS/[P_1444_][Tf_2_N]/POMS membranes showed a marked improvement over neat POMS, confirming the positive effect of IL incorporation. In contrast, the unmodified membranes (POMS and PEBA) displayed much lower E values, highlighting their limited ability to discriminate between the organic and aqueous phases.

These results demonstrate that IL inclusion enhances both the affinity of the membrane for organic compounds and its capacity to selectively retain them over water. The increased enrichment is primarily attributed to the reduction in water permeability combined with improved solubility of the target compounds within the IL phase [62].

As shown in Table 6, the base membranes (POMS and PEBA) exhibited the lowest separation factor values, confirming that, in the absence of an IL, transport does not effectively discriminate between organic compounds and water.

In contrast, the trilayer membranes showed substantial increases in separation factor, particularly PEBA/[Bmim][Tf_2_N]/PEBA and PEBA/[P_1444_][Tf_2_N]/PEBA, which displayed the highest ${\;\beta }_{i/w}$ values for hexanal, whereas POMS/[P_1444_][Tf_2_N]/POMS exhibited the highest ${\;\beta }_{i/w}$ value for linalool, indicating that the optimal IL-polymer combination depends on the specific target compound. This enhancement is attributed to the ability of ILs to modify membrane chemical affinity, promoting π–π and dipole–dipole interactions, and specific interactions with oxygenated functional groups.

Among the ILs, [Bmim][Tf_2_N] favored the transport of moderately polar compounds, whereas [P_1444_][Tf_2_N] enhanced the separation factors of more hydrophobic species. These trends indicate that separation efficiency depends not only on IL chemistry but also on the compatibility between the polymer matrix and the target compound [61].

The comparison of overall mass transfer coefficients in Table 7 allows identification of the main factors governing separation performance. For single-layer membrane, POMS exhibited the lowest $K_{L\;TC}$ values, reflecting the strong hydrophobicity of the polymer and its preference for volatile organic compounds [63].

PEBA membranes showed slightly higher $K_{L\;TC}$ values for most compounds, with hexanal and linalool presenting the highest mobility. Although PEBA is more polar, the coexistence of hydrophobic segments and intermolecular interactions may limit the diffusion of highly polar solutes such as ethanol or benzyl alcohol [64].

In trilayer membranes, POMS/[P_1444_][Tf_2_N]/POMS exhibited increased $K_{L\;TC}$ values compared with neat POMS, particularly for linalool and hexanal, indicating reduced diffusional resistance induced by the phosphonium IL. Conversely, POMS/Bmim][Tf_2_N]/POMS showed similar or slightly lower coefficients, suggesting stronger solute-IL interactions that limit mobility.

Among all configurations, PEBA/[P_1444_][Tf_2_N]/PEBA exhibited the most increased $K_{L\;TC}$ values, confirming its superior permeability and transport efficiency. In contrast, PEBA/[Bmim][Tf_2_N]/PEBA exhibited reduced coefficients, indicating that imidazolium-based ILs may impose steric or electrostatic constraints on diffusion [65].

Overall, linalool and hexanal consistently showed the highest mass transfer coefficients, while ethanol exhibited the lowest due to its strong affinity for water. The results confirm that PEBA/[P_1444_][Tf_2_N]/PEBA is the most efficient configuration under the evaluated conditions, and that phosphonium-based ILs provide a more favorable environment for diffusion than imidazolium-based counterparts [66,67].

### 3.4. Mass Transfer and Membrane Transport Analysis

The mass transfer analysis was incorporated to interpret the pervaporation results beyond the direct comparison of extraction percentages, fluxes, enrichment factors, and selectivity. Using Equation (5), the overall transport coefficients were calculated by combining the experimental transmembrane flux with the thermodynamic driving force of each compound (Table 8). For this purpose, the feed mole fraction, saturation vapor pressure at 30 °C, and activity coefficient at infinite dilution ($\gamma^{\infty }$, were considered. The $\gamma^{\infty }$, values were estimated using the NRTL model and are summarized in Table 9.

The mass transfer behavior of the evaluated compounds can be interpreted by analyzing the relationship between the overall mass transfer coefficients ($K_{overall}$), the activity coefficients at infinite dilution ($\gamma^{\infty }$), and the vapor pressures of the pure compounds ($P_{i}^{sat}$). These three parameters capture the thermodynamic driving force for transfer from the aqueous phase as well as the overall ability of the membrane system to transport each solute.

The calculated activity coefficients at infinite dilution reveal a strong positive deviation from ideality for most AFs in water. Ethanol exhibited the lowest value ($\gamma^{\infty }\approx 3.98$), while significantly larger values were obtained for the hydrophobic compounds, including hexanol ($\gamma^{\infty }\approx 1.11\times 10^{2}$), linalool ($\gamma^{\infty }\approx 1.35\times 10^{3}$), benzyl alcohol ($\gamma^{\infty }\approx 4.17\times 10^{2}$), and especially hexanal ($\gamma^{\infty }\approx 1.03\times 10^{4}$). These high values indicate a strong thermodynamic tendency of these compounds to escape the aqueous phase, which constitutes an important driving force for their transfer toward the membrane phase.

However, the results show that the magnitude of $K_{overall}$ is not directly proportional to $\gamma^{\infty }$. For example, hexanal presents one of the highest activity coefficients, indicating very low affinity for water, but its overall mass transfer coefficient is not the largest among the compounds studied. In contrast, benzyl alcohol consistently exhibits the highest $K_{overall}$ values across the evaluated membranes despite having a significantly lower $\gamma^{\infty }$ than hexanal. This observation indicates that the global transport process is not governed solely by the thermodynamic instability of the compound in water, but also by its interactions with the membrane and its ability to diffuse within the membrane phase.

A similar conclusion emerges when considering the vapor pressure of the compounds. Ethanol displays by far the highest vapor pressure at 30 °C ($P^{sat}\approx 10.47$ kPa), whereas the other AFs exhibit substantially lower values, typically below 1 kPa. If volatility were the dominant factor in controlling mass transfer, ethanol would be expected to present the highest global transport coefficient. Nevertheless, the experimental analysis shows that ethanol exhibits relatively modest $K_{overall}$ values compared with less volatile compounds such as benzyl alcohol, linalool, or hexanol. This clearly demonstrates that volatility alone does not determine the mass transfer performance of the system.

The observed behavior suggests that the transport of AFs in these membranes is governed by a combined effect of thermodynamic driving force and solute–membrane interactions. Highly hydrophobic compounds, even with moderate vapor pressures, tend to exhibit larger overall transport coefficients because they partition more favorably into the polymeric membrane or the ionic liquid phase and diffuse more efficiently through the membrane structure.

Overall, the comparison between $P^{sat}$, $\gamma^{\infty }$, and $K_{overall}$ indicates that the global mass transfer in the studied system cannot be explained solely by volatility or aqueous non-ideality. Instead, effective transport results from the interplay between the thermodynamic driving force from the aqueous phase and the affinity and mobility of the solute within the membrane phase.

In order to further interpret the transport mechanisms controlling the pervaporation process, a decomposition of the overall mass transfer coefficient into liquid-phase and membrane contributions was performed. However, the estimation of the liquid-phase mass transfer coefficient requires the evaluation of several transport properties, including density, viscosity, and diffusion coefficients of the solutes in water.

The density and viscosity of the solutions were assumed to be approximately equal to those of water at 30 °C due to the very low solute concentration (1000 ppm), which results in negligible deviations from the physical properties of pure water. Diffusion coefficients of the AFs in water ($D_{ab}$) were estimated using correlations commonly applied for dilute organic solutes in aqueous systems.

The calculated diffusion coefficients show some variability among the compounds due to differences in molecular size and structure. However, the magnitude of $D_{ab}$ remains within the same order, typically around $10^{-9\;}m^{2}$·$s^{-1}$. Consequently, although heavier molecules such as benzyl alcohol and linalool exhibit slightly lower diffusivities than smaller molecules such as ethanol, the differences are relatively modest. This indicates that diffusion in the aqueous phase is not expected to introduce drastic variations in the liquid-phase transport resistance.

Using the estimated transport properties, Reynolds, Schmidt, and Sherwood numbers were calculated for the rectangular flow channel of the membrane module. These values allowed the determination of the convective mass transfer coefficient in the liquid phase ($k_{L}$) for each compound.

To further interpret the transport mechanisms controlling pervaporation, the overall resistance was decomposed into liquid-phase and membrane contributions. Because the feed contained dilute AF compounds (1000 ppm), the density and viscosity of the solution were assumed to be close to those of water at 30 °C. The diffusion coefficients were estimated for dilute organic solutes in water, and the corresponding Reynolds, Schmidt, Sherwood, and liquid-phase mass transfer coefficients are shown in Table 2 in complementary documents.

Once the overall mass transfer coefficients were determined, their distribution between the liquid boundary layer (K_l_) and the membrane phase (K_m_) was evaluated for each compound, with the complete resistance distributions provided in the Supplementary Materials. Because K_l_ depends only on the hydrodynamic conditions and the diffusivity of each solute in water, it remained constant across membrane configurations, whereas K_m_ varied substantially depending on the polymer and IL identity. Under the hydrodynamic conditions employed in this study, transport was predominantly controlled by the membrane phase for all compounds, although the relative contribution of the liquid boundary layer increased for larger, less diffusive molecules such as linalool and hexanal.

The distribution of liquid-phase and membrane resistances was further examined for each individual membrane configuration (Supplementary Materials). Across all six configurations, the membrane phase consistently represented the dominant transport resistance, while the liquid boundary layer contributed only a minor fraction, particularly for water and small polar molecules such as ethanol. For the unmodified POMS- and PEBA-support membranes, this membrane-dominated hierarchy reflects the absence of an additional selective barrier, whereas incorporation of the ionic liquids modulated the relative balance between resistances without displacing the membrane phase as the primary limiting step.

The specific IL cation influenced this balance in a compound-dependent manner. The imidazolium-based [Bmim][Tf_2_N] promoted stronger interactions with aromatic and unsaturated compounds through its heterocyclic ring, moderately increasing the liquid-phase contribution for these solutes, whereas the bulkier, non-aromatic phosphonium-based [P_1444_][Tf_2_N] interacted mainly through non-specific dispersive forces, producing smaller shifts in the resistance distribution. In both cases, however, the membrane resistance remained dominant for the majority of the evaluated aroma compounds.

To compare the intrinsic transport capacity of each membrane configuration, membrane permeabilities were calculated for all aroma and fragrance compounds using the experimentally determined transmembrane fluxes, the corresponding driving force, and the effective membrane thickness. Unlike the overall flux, permeability allows the influence of membrane thickness and vapor-pressure-driven transport to be considered, providing a more direct comparison of the affinity and diffusional capacity of each membrane toward the evaluated compounds. The resulting permeability values are summarized in Table 10.

The calculated permeabilities confirm that transport through these membranes follows a sorption–diffusion mechanism. Benzyl alcohol showed the highest permeability in all membrane configurations, suggesting strong affinity toward the organophilic membrane environment. In contrast, water and ethanol presented the lowest permeabilities, confirming the limited sorption of these polar molecules in the hydrophobic polymer/ionic liquid phases.

Linalool, hexanol, and the hexenol isomers displayed intermediate-to-high permeabilities, which can be attributed to favorable partitioning into the membrane combined with diffusional limitations associated with molecular size and structure. Hexanal showed relatively modest permeability despite its high γ∞ value, reinforcing that a strong thermodynamic tendency to leave the aqueous phase does not necessarily translate into the highest membrane permeability. Therefore, the effective transport depends on the combined effects of feed-side driving force, membrane affinity, and diffusivity within the membrane structure. The membrane selectivity values relative to water for each compound and membrane configuration are summarized in Table 11.

From Table 11, the selectivity values demonstrate the strong influence of the ionic liquid phase on the separation performance. Although the POMS and PEBA support membranes already exhibited a certain preference for organic compounds over water, the incorporation of hydrophobic ILs substantially amplified this effect. This behavior is attributed to a synergistic effect between the hydrophobic polymer matrix and the IL microenvironment, which promotes preferential partitioning of AF compounds while suppressing water transport.

The enhancement was compound-dependent. Larger and more hydrophobic molecules, such as benzyl alcohol and linalool, exhibited the highest selectivity values, whereas smaller and more polar molecules, such as ethanol, showed more moderate improvements. Differences between the imidazolium and phosphonium ILs were also observed. The imidazolium cation can promote specific interactions with aromatic and unsaturated molecules through π–π and dispersion interactions, while the phosphonium cation behaves mainly as a bulky, highly hydrophobic medium governed by non-specific dispersive interactions. The pervaporation separation index (PSI) values calculated for each compound and membrane configuration are summarized in Table 12.

The pervaporation separation index (PSI) integrates flux and selectivity, providing a practical indicator of separation performance. In agreement with the permeability and selectivity results, the membranes containing ILs generally exhibited higher PSI values than the corresponding support membranes. The highest PSI values were obtained for linalool and benzyl alcohol, particularly in IL-containing configurations, indicating that their enhanced selectivity was accompanied by sufficiently high permeation fluxes.

Overall, the mass transfer analysis confirms that the superior performance of the trilayer membranes is not explained by a single physicochemical property. Instead, it results from the combined contribution of the thermodynamic driving force in the aqueous phase, preferential sorption into the organophilic membrane environment, and diffusion through the polymer/IL structure. Therefore, the rational selection of both the polymeric support and the IL cation is essential to optimize the recovery of AF compounds by pervaporation.

### 3.5. Comparison with Data from Literature

To contextualize the performance of the developed membranes, a comparative analysis was carried out with previously reported studies on pervaporation processes applied to the recovery of aromatic and volatile compounds from aqueous matrices. This comparison allows for identifying both convergences and divergences between the present results and those reported for conventional polymeric membranes, particularly PDMS and POMS systems.

As shown in Table 13, the total permeate and enrichment factor (E) values reported in the literature for the pervaporation of aroma compounds are, in most cases, lower than those obtained in the present study. Conventional single-layer membranes, such as PDMS, POMS, SBS composites, and commercial pervaporation membranes, typically exhibit total fluxes ranging from 10^−4^ to 10^−3^ kg h^−1^ m^−2^ and enrichment factors generally below 300 for compounds such as hexanol, hexanal, linalool, and hexenol isomers.

In contrast, the trilayer membranes incorporating gelled ILs developed in this work achieved transmembrane fluxes on the order of 10^−2^ kg h^−1^ m^−2^, together with enrichment factors exceeding 500 for linalool and 1450 for hexanal. These values represent a substantial improvement over conventional systems, even when compared with studies reporting high enrichment factors, which often occur at the expense of very low permeation fluxes.

This enhanced performance highlights the decisive role of the gelled IL phase, which acts as a selective and dynamic transport medium within the membrane structure. Unlike traditional polymer-only membranes, the IL layer provides an additional degree of freedom to modulate polarity, solubility, and molecular interactions, promoting preferential partitioning of aromatic compounds while simultaneously suppressing water transport.

Furthermore, the dual chemical character of ILs, comprising both polar and nonpolar domains, facilitates specific interactions with oxygenated functional groups present in aroma compounds, such as hydroxyl and aldehyde groups. This improves chemical compatibility between the polymer matrix and the solutes; the gelation of the IL ensures mechanical stability and prevents leakage, allowing the system to maintain high permeability without sacrificing selectivity.

It should further be noted that most of the literature values compiled in Table 13 do not report the pervaporation separation index (PSI), which limits a fully objective comparison based on total flux and enrichment factor alone. As shown in Table 12, the PSI values obtained in this study for linalool and benzyl alcohol in IL-containing configurations (up to 7.76 and 43.26, respectively) combine both high selectivity and sufficiently high permeation flux, a balance that is rarely achieved simultaneously in the conventional single-layer membranes summarized in Table 13.

Overall, the comparison with literature data confirms that the incorporation of gelled ILs into trilayer membrane architecture enables a favorable balance between flux and enrichment, overcoming the typical trade-off observed in conventional pervaporation membranes. This approach represents a significant advantage in the selective recovery of aroma compounds from aqueous streams and demonstrates the potential of IL-based composite membranes for high-efficiency pervaporation applications.

## 4. Conclusions

The gelation of [P_1444_][Tf_2_N] and [Bmim][Tf_2_N] with 12-hydroxystearic acid (12-HSA) produced a stable three-dimensional network that immobilized the ionic liquid within the intermediate layer of the trilayer membranes, preventing macroscopic leakage while preserving favorable transport properties.

Among the evaluated configurations, PEBA/[Bmim][Tf_2_N]/PEBA offered the most balanced overall performance, combining high recovery of hexanal, linalool, and E-2-hexen-1-ol with enhanced organic/water selectivity, whereas [P_1444_][Tf_2_N]-based membranes favored more hydrophobic compounds such as hexanal. IL incorporation substantially increased enrichment factors relative to the neat polymer membranes and simultaneously suppressed water transport, confirming that the gelled IL interlayer enhances both selectivity and mass-transfer control.

Overall, these results show that the rational selection of the polymer matrix and IL chemistry allows fine control over permeability and mass-transfer resistance, supporting gel-stabilized ionic liquid trilayer membranes as a promising strategy for the sustainable recovery of aroma compounds from agro-industrial effluents via pervaporation.

## Figures and Tables

**Table 1:** Properties of the compounds used.

Compound	Chemical Group	Boiling Point (°C)	Vapor Pressure (kPa at 25 °C)	Chemical Structure
Ethanol	Alcohol	78.37	5.95	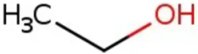
Hexanol	Alcohol	157.50	0.67	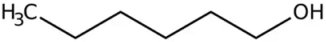
Linalool	Terpenic Alcohol	198.00	0.17	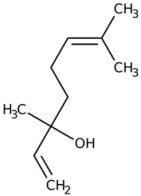
Benzyl Alcohol	Aromatic Alcohol	205.00	0.13	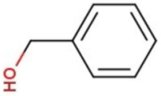
Hexanal	Aldehyde	131.00	0.13	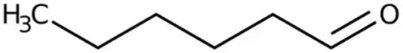
Z-3-hexen-1-ol	Unsaturated Alcohol	156.00	0.60	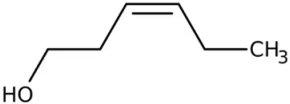
E-2-hexen-1-ol	Unsaturated Alcohol	157.00	0.58	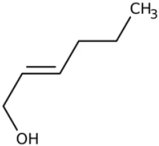

**Table 2:** ILs used as extractive phases in this study: nomenclature and structural formula.

Name	Nomenclature	Cation	Anion
1-butyl-3-methylimidazolium bis(trifluoromethylsulfonyl)imide	[Bmim][Tf_2_N]	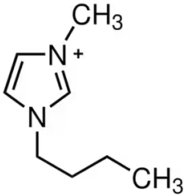	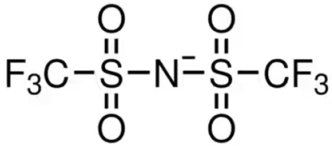
Tributyl-methyl-phosphonium bis(trifluoromethylsulfonyl)imide	[P_1444_][Tf_2_N]	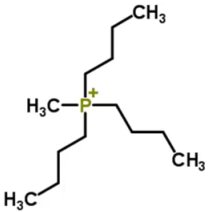	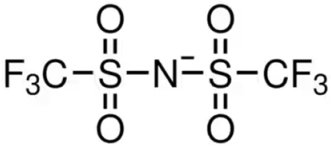

**Table 3:** Experimental parameters and variables used in the pervaporation system.

Membrane Type	Flow Rate (mL min^−1^)	Temperature (°C)	Vacuum Pressure (mbar)	Membrane Thickness (mm)
POMS	1000	30	500	0.17
PEBA	1000			0.27
POMS/[P_1444_][Tf_2_N]/POMS	100			0.34
	500			
	1000			
POMS/[Bmim][Tf_2_N]/POMS	100			0.34
	500			
	1000			
PEBA/[P_1444_][Tf_2_N]/PEBA	100			0.54
	500			
	1000			
PEBA/[Bmim][Tf_2_N]/PEBA	100			0.54
	500			
	1000			

**Table 4:** Transmembrane water fluxes at 1000 mL min^−1^ for each membrane.

Membrane	Time (h)	N (kg h^−1^ m^−2^)
POMS-support	6	0.069
PEBA-support	6	0.312
POMS/[P_1444_][Tf_2_N]/POMS	22	0.006
POMS/[Bmim][Tf_2_N]/POMS	12	0.039
PEBA/[P_1444_][Tf_2_N]/PEBA	17	0.023
PEBA/[Bmim][Tf_2_N]/PEBA	22	0.013

**Table 5:** Enrichment factor (*E*) of each compound through the different membranes at a feed flow rate of 1000 (mL min^−1^).

Membrane	Compounds						
	Hexanol	Benzyl Alcohol	Linalool	Ethanol	Hexanal	Z-3-hexen-1-ol	E-2-hexen-1-ol
POMS	33.959	13.887	215.619	9.108	687.586	28.564	50.438
PEBA	11.105	2.626	73.434	1.946	133.606	6.087	5.566
POMS/[P_1444_][Tf_2_N]/POMS	154.087	36.440	1061.237	22.972	829.405	46.943	56.431
POMS/[Bmim][Tf_2_N]/POMS	49.790	22.845	350.165	8.611	412.676	29.645	46.700
PEBA/[P_1444_][Tf_2_N]/PEBA	200.219	39.727	962.644	21.179	1625.986	62.143	68.730
PEBA/[Bmim][Tf_2_N]/PEBA	51.227	28.093	561.499	13.943	1454.574	67.221	73.690

**Table 6:** Experimental separation factor of each compound with respect to water (*β_i_*_/*w*_) in each membrane at feed flow rate of 1000 (mL min^−1^).

Membrane	Compounds						
	Hexanol	Benzyl Alcohol	Linalool	Ethanol	Hexanal	Z-3-hexen-1-ol	E-2-hexen-1-ol
POMS	35.288	14.149	240.931	9.214	793.215	29.433	53.289
PEBA	11.219	2.634	75.646	1.950	137.674	6.116	5.596
POMS/[P_1444_][Tf_2_N]/POMS	223.426	43.121	2653.093	25.652	1797.044	56.332	70.539
POMS/[Bmim][Tf_2_N]/POMS	53.748	23.724	421.994	8.743	495.211	30.861	49.813
PEBA/[P_1444_][Tf_2_N]/PEBA	241.133	41.800	1364.443	21.824	2403.631	66.196	74.109
PEBA/[Bmim][Tf_2_N]/PEBA	57.532	30.254	911.356	14.479	2662.989	74.460	87.294

**Table 7:** Overall mass transfer coefficient *K_L TC_* (m h^−1^) of each compound in the membranes, measured at a feed flow rate of 1000 (mL min^−1^).

Membrane	Compounds						
	Hexanol	Benzyl Alcohol	Linalool	Ethanol	Hexanal	Z-3-hexen-1-ol	E-2-hexen-1-ol
POMS	3.14 × 10^−3^	1.33 × 10^−3^	1.75 × 10^−2^	8.79 × 10^−4^	3.28 × 10^−2^	2.66 × 10^−3^	4.55 × 10^−3^
PEBA	3.46 × 10^−3^	8.71 × 10^−4^	1.99 × 10^−2^	6.47 × 10^−4^	2.60 × 10^−2^	1.96 × 10^−3^	1.81 × 10^−3^
POMS/[P_1444_][Tf_2_N]/POMS	4.25 × 10^−3^	1.07 × 10^−3^	2.32 × 10^−2^	6.79 × 10^−4^	1.78 × 10^−2^	1.37 × 10^−3^	1.64 × 10^−3^
POMS/[Bmim][Tf_2_N]/POMS	3.03 × 10^−3^	1.42 × 10^−3^	1.79 × 10^−2^	5.43 × 10^−4^	1.82 × 10^−2^	1.82 × 10^−3^	2.83 × 10^−3^
PEBA/[P_1444_][Tf_2_N]/PEBA	5.31 × 10^−3^	1.17 × 10^−3^	2.20 × 10^−2^	6.28 × 10^−4^	2.76 × 10^−2^	1.81 × 10^−3^	2.01 × 10^−3^
PEBA/[Bmim][Tf_2_N]/PEBA	1.91 × 10^−3^	1.08 × 10^−3^	1.62 × 10^−2^	5.38 × 10^−4^	2.85 × 10^−2^	2.49 × 10^−3^	2.69 × 10^−3^

**Table 8:** Feed composition, saturation vapor pressure, and infinite-dilution activity coefficients used in Equation (5).

Compound	$x_{i}$ (1000 ppm in Water)	$P_{i}^{sat}$ at 30 °C (Pa)	$\gamma^{\infty }$, (303 k)
Water	1.000000	4240	1.00
Ethanol	3.91 × 10^−4^	10,470	3.98
Hexanol	1.76 × 10^−4^	1009	111.4
Linalool	1.17 × 10^−4^	262	1351
Benzyl alcohol	1.67 × 10^−4^	196	16.66
Hexanal	1.80 × 10^−4^	171	10,278
Z-3-hexen-1-ol	1.80 × 10^−4^	792	111.4
E-2-hexen-1-ol	1.80 × 10^−4^	766	111.4

**Table 9:** The overall mass transfer coefficients calculated from Equation (5) (kg m^−2^ h^−1^ Pa^−1^).

Compound	POMS-SUPPORT	PEBA-SUPPORT	POMS/[P_1444_][Tf_2_N]/POMS	POMS/[Bmim][Tf_2_N]/POMS	PEBA/[P_1444_][TF_2_N]/PEBA	PEBA/[Bmim][Tf_2_N]/PEBA
Water	1.63 × 10^−5^	7.36 × 10^−5^	1.42 × 10^−6^	9.20 × 10^−6^	5.42 × 10^−6^	3.07 × 10^−6^
Ethanol	4.89 × 10^−5^	3.67 × 10^−5^	4.28 × 10^−5^	3.67 × 10^−5^	4.28 × 10^−5^	3.06 × 10^−5^
Hexanol	1.37 × 10^−4^	1.62 × 10^−4^	1.37 × 10^−4^	1.57 × 10^−4^	2.38 × 10^−4^	8.10 × 10^−5^
Linalool	1.92 × 10^−4^	2.23 × 10^−4^	2.14 × 10^−4^	1.90 × 10^−4^	2.28 × 10^−4^	1.92 × 10^−4^
Benzyl alcohol	2.38 × 10^−3^	1.65 × 10^−3^	2.02 × 10^−3^	2.75 × 10^−3^	2.20 × 10^−3^	1.83 × 10^−3^
Hexanal	3.32 × 10^−5^	2.97 × 10^−5^	2.19 × 10^−5^	2.44 × 10^−5^	3.45 × 10^−5^	3.39 × 10^−5^
Z-3-hexen-1-ol	1.33 × 10^−4^	9.52 × 10^−5^	7.62 × 10^−5^	1.02 × 10^−4^	9.52 × 10^−5^	8.89 × 10^−5^
E-2-hexen-1-ol	2.47 × 10^−4^	1.08 × 10^−4^	9.52 × 10^−5^	1.65 × 10^−4^	1.14 × 10^−4^	1.52 × 10^−4^

**Table 10:** Membrane permeability calculated for each compound and membrane configuration (kg m^−1^ h^−1^ Pa^−1^). [P_1444_] and [Bmim] correspond to [P_1444_][Tf_2_N] and [Bmim][Tf_2_N], respectively.

Compound	POMS-SUPPORT	PEBA-SUPPORT	POMS/[P_1444_][Tf_2_N]/POMS	POMS/[Bmim][Tf_2_N]/POMS	PEBA/[P_1444_][Tf_2_N]/PEBA	PEBA/[Bmim][Tf_2_N]/PEBA
Water	2.77 × 10^−9^	2.00 × 10^−8^	4.83 × 10^−10^	3.13 × 10^−9^	2.93 × 10^−9^	1.66 × 10^−9^
Ethanol	8.48 × 10^−9^	1.01 × 10^−8^	1.48 × 10^−8^	1.27 × 10^−8^	2.35 × 10^−8^	1.67 × 10^−8^
Hexanol	2.58 × 10^−8^	4.92 × 10^−8^	5.16 × 10^−8^	6.02 × 10^−8^	1.54 × 10^−7^	4.64 × 10^−8^
Linalool	4.62 × 10^−8^	9.35 × 10^−8^	1.29 × 10^−7^	1.08 × 10^−7^	2.19 × 10^−7^	1.47 × 10^−7^
Benzyl alcohol	4.25 × 10^−7^	4.61 × 10^−7^	7.16 × 10^−7^	9.89 × 10^−7^	1.24 × 10^−6^	1.03 × 10^−6^
Hexanal	8.28 × 10^−9^	1.12 × 10^−8^	9.50 × 10^−9^	1.12 × 10^−8^	2.87 × 10^−8^	2.75 × 10^−8^
Z-3-hexen-1-ol	2.43 × 10^−8^	2.70 × 10^−8^	2.70 × 10^−8^	3.67 × 10^−8^	5.41 × 10^−8^	5.04 × 10^−8^
E-2-hexen-1-ol	4.79 × 10^−8^	3.09 × 10^−8^	3.40 × 10^−8^	6.14 × 10^−8^	6.54 × 10^−8^	8.89 × 10^−8^

**Table 11:** Membrane selectivity relative to water (*α_i_*_,*w*_) for each compound and membrane configuration.

Compound	POMS-SUPPORT	PEBA-SUPPORT	POMS/[P_1444_][Tf_2_N]/POMS	POMS/[Bmim][Tf_2_N]/POMS	PEBA/[P_1444_][Tf_2_N]/PEBA	PEBA/[Bmim][Tf_2_N]/PEBA
Water	1.00	1.00	1.00	1.00	1.00	1.00
Ethanol	3.06	0.51	30.64	4.06	8.02	10.06
Hexanol	9.31	2.46	106.83	19.23	52.56	27.95
Linalool	16.68	4.68	267.08	34.50	74.74	88.55
Benzyl alcohol	153.43	23.05	1482.40	315.97	423.21	620.48
Hexanal	2.99	0.56	19.67	3.58	9.80	16.57
Z-3-hexen-1-ol	8.77	1.35	55.90	11.73	18.46	30.36
E-2-hexen-1-ol	17.29	1.55	70.39	19.62	22.32	53.55

**Table 12:** Pervaporation separation index (PSI) for each compound and membrane configuration.

Compound	POMS-SUPPORT	PEBA-SUPPORT	POMS/[P_1444_][Tf_2_N]/POMS	POMS/[Bmim][Tf_2_N]/POMS	PEBA/[P_1444_][Tf_2_N]/PEBA	PEBA/[Bmim][Tf_2_N]/PEBA
Water	0	0	0	0	0	0
Ethanol	0.20291	−0.166012	0.865488	0.196452	0.37557	0.351528
Hexanol	0.818535	0.494648	3.090236	1.170366	2.75846	1.04566
Linalool	1.54448	1.246784	7.769536	2.1507	3.94509	3.39694
Benzyl alcohol	15.01436	7.47054	43.25688	20.22107	22.58824	24.03582
Hexanal	0.196015	−0.149072	0.545164	0.165636	0.4708	0.604116
Z-3-hexen-1-ol	0.765345	0.11858	1.60308	0.688866	0.93411	1.139168
E-2-hexen-1-ol	1.604565	0.18634	2.026188	1.195404	1.14062	2.03894

**Table 13:** Extraction of volatile organic compounds by the pervaporation process.

Compounds	Source	Membrane	Total Flux (kg h^−1^ m^−2^)	Enrichment Factor (E)	Reference
Ethanol	Model apple juice solution	PDMS	1.07 × 10^−1^	44–125	[68]
Hexanol					
Hexanal					
Hexanol	Muscatel wine	POMS	1 × 10^−3^	10–160	[69]
Linalool	Cranberry juice model solution	PDMS	-	231	[70]
Hexanol				203	
Z-3-hexen-1-ol	Tea extract	POMS	3 × 10^−3^	120	[71]
Hexanol	Diluted flavors systems	POMS	6.5 × 10^−2^	40	[72]
E-2-hexen-1-ol	Clarified kiwi juice	Pervap 1060	1.3 × 10^−1^	20	[73]
Hexanol				50	
E-2-hexen-1-ol	Kiwi juice	SBS composite	1 × 10^−3^	55	[74]
Hexanol				80	
Hexanal	Orange juice	PDMS	1 × 10^−4^	6	[75]
Linalool			3.5 × 10^−3^	12	
E-2-hexen-1-ol	Cranberry juice	PDMS	3.78 × 10^−8^	120.6	[76]
Hexanol			1.49 × 10^−7^	236.9	
Linalool			6.2 × 10^−9^	5.5	
Z-3-hexen-1-ol			5.15 × 10−8	27.4	
Benzyl alcohol			1.05 × 10−8	4.2	
Ethanol			2.51 × 10−3	13.7	
Hexanol	Pomegranate juice	PDMS	4 × 10^−4^	15	[77]
Hexanol	Grape must	PDMS	0.1	1–2	[78]
Hexanal					
Benzyl alcohol					
Hexanal	Hydrolate of plum, apple, blackcurrant, and cherry	Pervap ECO 001BP	8 × 10^−3^	3678	[79]
Hexanol	Model solution	POMS/[P1444][Tf2N]/POMS	2.92 × 10^−2^	154.087	This study
Benzyl alcohol				36.44	
Linalool				1061.237	
Ethanol				22.972	
Hexanal				829.405	
Z-3-hexen-1-ol				46.943	
E-2-hexen-1-ol				56.431	
Hexanol	Model solution	POMS/[Bmim][Tf2N]/POMS	6.42 × 10^−2^	49.79	This study
Benzyl alcohol				22.845	
Linalool				350.165	
Ethanol				8.611	
Hexanal				412.676	
Z-3-hexen-1-ol				29.645	
E-2-hexen-1-ol				46.7	
Hexanol	Model solution	PEBA/[P1444][Tf2N]/PEBA	5.35 × 10^−2^	200.219	This study
Benzyl alcohol				39.727	
Linalool				962.644	
Ethanol				21.179	
Hexanal				1625.986	
Z-3-hexen-1-ol				62.143	
E-2-hexen-1-ol				68.73	
Hexanol	Model solution	PEBA/[Bmim][Tf2N]/PEBA	3.88 × 10^−2^	51.227	This study
Benzyl alcohol				28.093	
Linalool				561.499	
Ethanol				13.943	
Hexanal				1454.574	
Z-3-hexen-1-ol				67.221	
E-2-hexen-1-ol				73.69	

## Data Availability

The data presented in this study are available upon reasonable request from the corresponding author (gmerlet@udec.cl).

## Supplementary Materials

The following supporting information can be downloaded at: https://www.mdpi.com/article/10.3390/membranes16080274/s1, Table S1: Transmembrane flux (N, kg m^−2^ h^−1^) at a feed flow rate of 1000 mL min^−1^; Table S2: Transport properties and hydrodynamic parameters used for the liquid-phase mass transfer analysis; Table S3: Distribution of resistance-in-series for water; Table S4: Distribution of resistance-in-series for ethanol; Table S5: Distribution of resistance-in-series for hexanol; Table S6: Distribution of resistance-in-series for linalool; Table S7: Distribution of resistance-in-series for benzyl alcohol; Table S8: Distribution of resistance-in-series for hexanal; Table S9: Distribution of resistance-in-series for Z-3-hexen-1-ol; Table S10: Distribution of resistance-in-series for E-2-hexen-1-ol.
