# Supplementary material for: Development of Polymer/Ionic Liquid/Polymer Trilayer Membranes for the Recovery of Blueberry Aroma and Fragrance Compounds from a Model Aqueous Solution by Pervaporation
Source: Membranes (Basel). 2026 Aug 17;16(8):274. doi: 10.3390/membranes16080274 (PMC13515546; doi:10.3390/membranes16080274)
Supplement: Supplementary file 1 [file membranes-16-00274-s001.zip › membranes-4403248-supplementary.pdf]

Supplementary Information

Table S1. transmembrane flux (N kg/m^{2}h) to 1000 mL/min

Compuesto	POMS-support	PEBA-support	POMS/[P1444][Tf2N]/POMS	POMS/[Bmim][Tf2N]/POMS	PEBA/[P1444][Tf2N]/PEBA	PEBA/[Bmim][Tf2N]/PEBA
Water	6.90×10^{-2}	3.12×10^{-1}	6.00×10^{-3}	3.90×10^{-2}	2.30×10^{-2}	1.30×10^{-2}
Hexanol	2.7×10^{-3}	3.2×10^{-3}	2.7×10^{-3}	3.1×10^{-3}	4.7×10^{-3}	1.6×10^{-3}
Benzyl alcohol	1.3×10^{-3}	9.0×10^{-4}	1.1×10^{-3}	1.5×10^{-3}	1.2×10^{-3}	1.0×10^{-3}
Linalool	8.1×10^{-3}	9.4×10^{-3}	9.0×10^{-3}	8.0×10^{-3}	9.6×10^{-3}	8.1×10^{-3}
Ethanol	8.0×10^{-4}	6.0×10^{-4}	7.0×10^{-4}	6.0×10^{-4}	7.0×10^{-4}	5.0×10^{-4}
Hexanal	1.06×10^{-2}	9.5×10^{-3}	7.0×10^{-3}	7.8×10^{-3}	1.10×10^{-2}	1.08×10^{-2}
Z-3-hexen-1-ol	2.1×10^{-3}	1.5×10^{-3}	1.2×10^{-3}	1.6×10^{-3}	1.5×10^{-3}	1.4×10^{-3}
E-2-hexen-1-ol	3.9×10^{-3}	1.7×10^{-3}	1.5×10^{-3}	2.6×10^{-3}	1.8×10^{-3}	2.4×10^{-3}

Table S2. Transport properties and hydrodynamic parameters used for the liquid-phase mass transfer analysis.

**Compound**	**ρ (kg m^{-3})**	**μ (Pa s)**	**D (m^2 s^{-1})**	**Re**	**Sc**	**Sh**	**kL (m h^{-1})**
Water	996.0	0.00080	2.60×10^{-9}	703.39	308.93	42.11	0.06922
Ethanol	996.0	0.00080	1.25×10^{-9}	703.39	642.57	52.38	0.04139
Hexanol	996.0	0.00080	0.75×10^{-9}	703.39	1070.95	61.15	0.02899
Linalool	996.0	0.00080	0.60×10^{-9}	703.39	1338.69	65.46	0.02483
Benzyl alcohol	996.0	0.00080	0.70×10^{-9}	703.39	1147.45	62.45	0.02763
Hexanal	996.0	0.00080	0.90×10^{-9}	703.39	892.46	57.85	0.03291
Z-3-hexen-1-ol	996.0	0.00080	0.80×10^{-9}	703.39	1004.02	59.96	0.03032
E-2-hexen-1-ol	996.0	0.00080	0.80×10^{-9}	703.39	1004.02	59.96	0.03032

Table S3. The distribution of resistance-in-series for Water.

Membrane	KL	KM	RL (%)	RM (%)
POMS-support	0.01625	0.00001632	0.10	99.90
PEBA-support	0.01625	0.00007393	0.45	99.55
POMS/[P1444][Tf2N]/POMS	0.01625	0.00000142	0.01	99.99
POMS/[Bmim][Tf2N]/POMS	0.01625	0.00000921	0.06	99.94
PEBA/[P1444][Tf2N]/PEBA	0.01625	0.00000542	0.03	99.97
PEBA/[Bmim][Tf2N]/PEBA	0.01625	0.00000307	0.02	99.98

Table S4. The distribution of resistance-in-series for Ethanol.

Membrane	KL	KM	RL (%)	RM (%)
POMS-support	0.00253	0.00004986	1.93	98.07
PEBA-support	0.00253	0.00003724	1.45	98.55
POMS/[P1444][Tf2N]/POMS	0.00253	0.00004354	1.69	98.31
POMS/[Bmim][Tf2N]/POMS	0.00253	0.00003724	1.45	98.55
PEBA/[P1444][Tf2N]/PEBA	0.00253	0.00004354	1.69	98.31
PEBA/[Bmim][Tf2N]/PEBA	0.00253	0.00003097	1.21	98.79

Table S5. The distribution of resistance-in-series for **Hexanol**

Membrane	KL	KM	RL (%)	RM (%)
POMS-support	0.00146	0.0001518	9.42	90.58
PEBA-support	0.00146	0.0001823	11.10	88.90
POMS/[P1444][Tf2N]/POMS	0.00146	0.0001518	9.42	90.58
POMS/[Bmim][Tf2N]/POMS	0.00146	0.000177	10.81	89.19
PEBA/[P1444][Tf2N]/PEBA	0.00146	0.0002856	16.36	83.64
PEBA/[Bmim][Tf2N]/PEBA	0.00146	0.00008584	5.55	94.45

Table S6. The distribution of resistance-in-series for **Linalool**

Membrane	KL	KM	RL (%)	RM (%)
POMS-support	0.000597	0.000272	31.30	68.70
PEBA-support	0.000597	0.0003464	36.72	63.28

POMS/[P1444][Tf2N]/POMS	0.000597	0.0003808	38.93	61.07
POMS/[Bmim][Tf2N]/POMS	0.000597	0.0003163	34.63	65.37
PEBA/[P1444][Tf2N]/PEBA	0.000597	0.0004065	40.50	59.50
PEBA/[Bmim][Tf2N]/PEBA	0.000597	0.000272	31.30	68.70

Table S7. The distribution of resistance-in-series for **Benzyl alcohol**

Membrane	KL	KM	RL (%)	RM (%)
POMS-support	0.0505	0.002503	4.72	95.28
PEBA-support	0.0505	0.001706	3.27	96.73
POMS/[P1444][Tf2N]/POMS	0.0505	0.002106	4.00	96.00
POMS/[Bmim][Tf2N]/POMS	0.0505	0.002909	5.45	94.55
PEBA/[P1444][Tf2N]/PEBA	0.0505	0.002301	4.36	95.64
PEBA/[Bmim][Tf2N]/PEBA	0.0505	0.001903	3.63	96.37

Table S8. The distribution of resistance-in-series for Water **Hexanal**

Membrane	KL	KM	RL (%)	RM (%)
POMS-support	0.000104	0.00004873	31.91	68.09
PEBA-support	0.000104	0.00004149	28.52	71.48
POMS/[P1444][Tf2N]/POMS	0.000104	0.00002795	21.18	78.82
POMS/[Bmim][Tf2N]/POMS	0.000104	0.00003299	24.08	75.92
PEBA/[P1444][Tf2N]/PEBA	0.000104	0.00005312	33.81	66.19
PEBA/[Bmim][Tf2N]/PEBA	0.000104	0.000051	32.90	67.10

Table S9. The distribution of resistance-in-series for **Z-3-hexen-1-ol**

Membrane	KL	KM	RL (%)	RM (%)
POMS-support	0.0019	0.0001429	6.99	93.01
PEBA-support	0.0019	0.0001002	5.01	94.99
POMS/[P1444][Tf2N]/POMS	0.0019	0.00007944	4.01	95.99
POMS/[Bmim][Tf2N]/POMS	0.0019	0.0001079	5.37	94.63
PEBA/[P1444][Tf2N]/PEBA	0.0019	0.0001002	5.01	94.99
PEBA/[Bmim][Tf2N]/PEBA	0.0019	0.0000933	4.68	95.32

Table S10. The distribution of resistance-in-series for **E-2-hexen-1-ol**

Membrane	KL	KM	RL (%)	RM (%)
POMS-support	0.00197	0.0002818	12.52	87.48
PEBA-support	0.00197	0.0001146	5.50	94.50
POMS/[P1444][Tf2N]/POMS	0.00197	0.0001	4.83	95.17
POMS/[Bmim][Tf2N]/POMS	0.00197	0.0001805	8.39	91.61
PEBA/[P1444][Tf2N]/PEBA	0.00197	0.0001211	5.79	94.21
PEBA/[Bmim][Tf2N]/PEBA	0.00197	0.0001647	7.72	92.28
